# Membrane Interfacial Organization Determines the Functional Performance of Liposomal Linezolid

**DOI:** 10.3390/pharmaceutics18080994

**Published:** 2026-08-11

**Authors:** Vadim Avdeev, Ilya Kolmogorov, Tatyana Tyulkova, Galina Mozhokina, Anastasia Samoilova, Anastasia Gaida, Anna Skuredina, Natalia Belogurova, Natalia Klyachko, Alexey Doroshenko, Irina Le-Deygen, Irina Vasilieva

**Affiliations:** 1 Federal State Institution National Medical Research Center of Tuberculosis and Infectious Diseases, Ministry of Health, Moscow 127473, Russia; 2 Department of Chemistry, Lomonosov Moscow State University, Moscow 119991, Russia

**Keywords:** tuberculosis, liposome, linezolid, ATR-FTIR spectroscopy, drug delivery

## Abstract

**Background:** Despite extensive development of liposomal antibiotics, the structural determinants governing their stability, release, and biological activity remain poorly understood. This study investigated how the cholesterol content and drug-to-lipid ratio affect membrane organization and thereby determine the physicochemical and biological properties of linezolid-loaded liposomes. **Methods:** Nine liposomal formulations, varying in their cholesterol content (10–30 wt%) and drug-to-lipid ratios (1–5%), were prepared by thin-film hydration. Membrane organization was analyzed by ATR-FTIR spectroscopy and principal component analysis. Liposomes were further characterized by particle size, ζ-potential, encapsulation efficiency, storage stability, in vitro release in phosphate buffer with and without bovine serum albumin, antibacterial activity against *B. subtilis*, and antimycobacterial activity in an ex vivo PBMC-derived *Mycobacterium tuberculosis* granuloma model. **Results:** The cholesterol content and drug-to-lipid ratio markedly altered membrane interfacial organization, particularly the hydration of the carbonyl and phosphate regions. These structural changes correlated with differences in storage stability, protein-responsive release, and antibacterial activity. Functional behavior was non-monotonic, as 30-L showed the highest overall storage stability, while the apparent release depended jointly on the cholesterol content, drug loading, and medium. BSA altered the composition-dependent release pattern instead of producing a uniform effect. In the exploratory granuloma model, the formulations 10-S, 10-L, and 30-M reduced *M. tuberculosis* CFU by >99%, whereas free linezolid produced approximately 60% inhibition. **Conclusions:** Membrane interfacial organization is a key determinant of the functional performance of liposomal linezolid, establishing a structure–property–function relationship that provides a mechanistic basis for the rational design of liposomal antibiotic delivery systems for tuberculosis therapy.

## 1. Introduction

Tuberculosis (TB), caused by *Mycobacterium tuberculosis* (Mtb), remains a major cause of infectious disease morbidity and mortality worldwide, with drug-resistant forms continuing to present a substantial therapeutic challenge [1]. The persistence of Mtb within macrophages [2] and structurally heterogeneous granulomatous lesions complicates drug delivery to infected compartments and contributes to the need for prolonged multidrug treatments. Linezolid (Lzd), the first clinically introduced oxazolidinone, is currently included in several World Health Organization-recommended regimens for multidrug- and rifampicin-resistant TB [3]. However, prolonged linezolid administration is frequently limited by exposure-dependent adverse effects, particularly myelosuppression, thrombocytopenia, and peripheral or optic neuropathy [4]. Clinical pharmacokinetic and toxicodynamic studies have demonstrated a relationship between systemic linezolid exposure, treatment duration, and platelet suppression [5].

Liposomal encapsulation in liposomal vesicle offers a potential strategy for modifying the distribution and release of linezolid while preserving its antibacterial activity [6,7,8]. Liposomes can be internalized by macrophages and may therefore facilitate drug delivery to an important intracellular niche of Mtb. Pulmonary administration may additionally increase local linezolid exposure in the lungs while reducing the systemic drug burden [9]. Given the established relationship between systemic linezolid exposure and hematological toxicity, such an approach could potentially improve tolerability, although this possibility requires direct pharmacokinetic and toxicological confirmation [5,10].

The functional performance of liposomal formulations depends on the relationship between lipid composition, drug loading, membrane organization, colloidal stability, and release behavior. Premature leakage may diminish the advantages of encapsulation, whereas excessive drug retention may restrict the availability of the active compound at the site of infection. Among the principal formulation variables, the cholesterol content and drug-to-lipid ratio (DLR) are particularly important, because both can alter the physicochemical state of the lipid bilayer.

The influence of cholesterol on phospholipid membrane properties is well-established [11]. Classical permeability studies have demonstrated that cholesterol incorporation modifies the transport of ions and small hydrophilic solutes through phospholipid membranes and generally reduces permeability in liquid–crystalline states [12]. Cholesterol also affects lipid packing, thermotropic phase behavior, interfacial hydration, and the organization of both polar and hydrophobic regions of phospholipid bilayers. FTIR-based studies have established the sensitivity of carbonyl- and phosphate-associated absorption bands to lipid hydration and conformational states [13], whereas spectroscopic and calorimetric investigations have demonstrated cholesterol-dependent changes in the phase organization of phosphatidylethanolamine- and phosphatidylcholine-containing membranes [14,15].

Importantly, the effect of cholesterol depends on the phospholipid composition, acyl-chain saturation, temperature, cholesterol fraction, and physicochemical properties of the incorporated drug. A systematic comparison of DMPC-, DPPC-, and DSPC-based liposomes containing 0–50 mol% cholesterol have demonstrated that both formulation stability and drug release depended on the combination of the phospholipid type, cholesterol content, and encapsulated compound; a phospholipid-to-cholesterol ratio of 70:30 mol/mol provided a favorable balance for the systems examined [16]. More recent measurements of membrane interfacial water showed that cholesterol enhanced acyl-chain packing and water ordering in saturated DPPC and sphingomyelin monolayers, whereas substantially weaker changes were observed in unsaturated DOPC membranes [17]. These observations are particularly relevant to soy lecithin formulations, which contain a heterogeneous mixture of predominantly unsaturated phospholipids and therefore cannot be assumed to reproduce the behavior of single-component saturated phosphatidylcholine membranes.

Published drug release studies have further demonstrated that cholesterol does not act as a universal membrane-sealing additive whose effect can be predicted independently of the incorporated drug. For azithromycin-loaded liposomes, increasing the phospholipid-to-cholesterol molar ratio from 2:1 to 4:1 increased the fraction of drug released within 24 h from 4.9% to 22%, quantitatively demonstrating the strong effect of cholesterol content on antibiotic retention [18]. In other systems, increasing cholesterol content reduced drug release, whereas the magnitude and direction of this effect varied with the phospholipid structure and drug localization [16]. Thus, cholesterol content alone is insufficient to predict the release properties of a specific liposomal drug formulation.

DLR represents a second major determinant of liposomal performance. The encapsulated compound may interact with phospholipid headgroups, carbonyl groups, or hydrocarbon chains and thereby modify bilayer hydration, packing, phase transitions, and curvature. In levofloxacin-loaded anionic liposomes, increasing the DLR reduced acyl-chain mobility and altered the hydration of lipid carbonyl and phosphate groups, indicating loading-dependent changes in membrane organization [19]. The relationship between DLR and release is likewise well-documented: in DSPC/cholesterol liposomes, increasing the doxorubicin-to-lipid mass ratio from 0.05 to 0.39 prolonged the in vitro release half-life by more than six-fold [20]. These findings indicate that cholesterol content and drug loading should not be considered independent variables, because the functional effect of cholesterol may depend on the amount and interfacial localization of the incorporated drug.

In the present study, a two-dimensional formulation matrix was used in which the cholesterol content and linezolid loading were varied independently. Liposomes containing 10, 20, or 30 wt% cholesterol relative to the total lipid mass were prepared at three drug loadings, yielding nine formulations. Their properties were evaluated using particle-size and ζ-potential measurements, FTIR analysis of membrane molecular organization, encapsulation efficiency, storage and freeze–thaw stability, dialysis-based linezolid release/transfer in protein-free and BSA-containing media, and antibacterial activity. The study aimed to determine how the cholesterol content and DLR jointly shape the interfacial organization of linezolid-loaded membranes, and how these structural differences are reflected in colloidal stability, drug retention, release behavior, and biological performance.

## 2. Materials and Methods

### 2.1. Materials

Linezolid (Lzd), soy lecithin (Lec), cholesterol (Chol), Triton X-100, glucose, mannose, L-glutamine, penicillin/streptomycin, non-essential amino acids, vitamins, and sodium pyruvate were obtained from Sigma Aldrich, St. Louis, MO, USA; tablets for the preparation of sodium phosphate buffer solution with pH 7.4 were from Pan-Eco, Moscow, Russia; and chloroform was from Khimmedservis LLC, Moscow, Russia. Dialysis bags with a cut-off molecular weight of 12–14 kDa were obtained from Orange Scientific, Braine-l’Alleud, Belgium. RPMI 1640 medium and 10% fetal bovine serum (FBS) were from Thermo Fisher Scientific, Waltham, MA, USA.

### 2.2. Liposome Preparation

Thin-film hydration followed by the ultrasonication technique were used to prepare the liposomes. Briefly, the chloroform solution of lipids (Lec, Chol all 25 mg/mL) in the desired ratios were transferred to a round bottom flask, and the solvent was removed using a vacuum rotary evaporator at a temperature below 55 °C. The resulting thin film was dispersed in a 0.01 M sodium phosphate buffer, pH 7.4, according to the following procedure. Firstly, the flask with buffer solution was exposed to an ultrasonic bath (37 kHz) for 5 min; after that, the nontransparent suspension was transferred to a plastic tube and then sonicated (22 kHz) for 600 s (2 × 5 min) in a continuous mode with constant cooling with a 4710 “Cole-Parmer Instrument” disperser (Cole-Parmer Instrument Co., Chicago, IL, USA).

### 2.3. Liposomal Form of Linezolid (LLzd) Preparation

To obtain liposomal forms of linezolid (LLzd), the same procedure was applied with the following changes: a thin film of lipids was dispersed within a 0.01 M sodium phosphate buffer solution, pH 7.4, containing linezolid (0.1 mg/mL). The unloaded drug was separated by dialysis against a 0.01 M sodium phosphate buffer solution, pH 7.4 (Orange Scientific, Braine-l’Alleud, Belgium; MW cut-off 12–14 kDa) for 120 min at 4 °C.

### 2.4. Encapsulation Efficiency and Drug–Lipid Mass Ratio Calculation

After dialysis, the liposomal dispersion was recovered from the donor compartment, and the vesicles were disrupted with 1% (*v*/*v*) Triton X-100 to release the encapsulated drug. Linezolid was quantified directly in the disrupted liposomal dispersion by UV spectroscopy using its characteristic absorption band at 250 nm and the corresponding calibration curve (Figure S1).

The mass of encapsulated linezolid was calculated from its measured concentration and the recovered volume of the liposomal dispersion. The drug-to-lipid mass ratio (*DLR*) was calculated according to Equation (1):

(1) $$DLR=\frac{m_{loaded\;linezolid}}{m_{lipids}}\;$$

The encapsulation efficiency (EE) of linezolid was determined using the material balance according to Equation (2):

(2) $$Encapsulation\;efficiency=\;\frac{\nu {\left(Linezolid\right)}_{loaded}}{\nu {\left(Linezolid\right)}_{total}}\times 100\%\;\;\;$$

### 2.5. Stability Studies

LLzd stability was studied during storage at +4 °C and −20 °C. Testing at −20 °C was performed in three ways. The first was simple freezing. The second involved pre-freezing in liquid nitrogen and then transferring the sample to a freezer at −20 °C. The third involved the use of mannose and glucose as cryoprotectants. The cryoprotectant solution was added to the liposome suspension in a 1:1 (*v*/*v*) ratio. For a 100 µL aliquot of liposomes (5 mg/mL lipid), 100 µL of the 2× cryoprotectant stock solution was added. This yielded a final concentration of 5% (*w*/*v*) 50 mg/mL for the cryoprotectant and 2.5 mg/mL for the lipid. The sample was gently mixed and incubated at room temperature for 15–30 min to allow equilibration. The liposome–cryoprotectant mixture was then subjected to simple freezing. After storage for the selected period, the samples were slowly defrosted and brought to room temperature, after which they were subjected to instrumental analysis methods. A corresponding sample was prepared for each storage period. Repeated freezing or refrigeration was not performed.

### 2.6. Nanoparticle Tracking Analysis (NTA)

Nanoparticle tracking analysis (NTA) was conducted utilizing the Nano Sight NS500 instrument manufactured by NanoSight Ltd. (Malvern Panalytical, Malverrn, UK), equipped with a 532 nm green laser and NTA software version 3.4 Build 3.4.003, according to the previously published methodology [21]. The instrument was pre-calibrated with monodisperse polystyrene microspheres of 100 nm diameter (Macklin Chemicals, Shanghai, China). Samples of liposomes were diluted with Milli-Q purified water to achieve a concentration of approximately 10^8^ particles per mL.

Three independently prepared liposome batches were analyzed for each formulation. Separate aliquots from the same batches were used for DLS and NTA immediately after preparation. Before analysis, the samples were gently homogenized, and no visible sedimentation or precipitation was observed. For each independently prepared batch, five videos were acquired. These five recordings were treated as technical acquisitions and averaged to obtain one NTA value for each independent batch. The arithmetic mean of hydrodynamic diameter, derived from the number-based particle-size distribution, was reported.

### 2.7. Dynamic Light Scattering (DLS)

DLS was used to determine the cumulant-derived Z-average hydrodynamic diameter and polydispersity index (PDI) of the liposomal formulations. Measurements were performed using a Zetasizer Nano ZS equipped with a 4 mW He–Ne laser, operating at 633 nm (Malvern Panalytical, UK) at 22 °C and using Zetasizer software version 3.30. The Z-average diameter was obtained by cumulant analysis of the intensity autocorrelation function.

Three independently prepared liposome batches were analyzed for each formulation. Separate aliquots from the same batches were used for DLS and NTA at the same post-preparation time point. Before measurement, the samples were gently homogenized, and no visible sedimentation or precipitation was observed. For each independent batch, 3 consecutive DLS acquisitions were performed and averaged to obtain one value for that batch. The values reported in the manuscript represent the mean ± SD calculated across the three independently prepared batches.

### 2.8. UV–Vis Spectroscopy

UV–Vis spectra were obtained from a UV–Vis spectrometer AmerSharm Biosciences UltraSpec 2100 pro (Holliston, MA, USA) in a 1 mL quartz cuvette from Hellma Analytics (Müllheim, Germany). The main characteristic peaks of linezolid were observed at 250 nm, correspondingly.

### 2.9. ATR-FTIR Spectroscopy

FTIR spectroscopic analyses were performed on a Bruker Tensor 27 spectrometer from Bruker (Ettlingen, Germany), which included a liquid-nitrogen-cooled MCT detector, a temperature control unit supplied by Huber (Offenburg, Germany), and an attenuated total reflectance accessory with a zinc selenide (ZnSe) single-reflection crystal. Spectra of the samples (volume 40–50 µL) were acquired in triplicate, consisting of 70 scans each, over the wavenumber range of 3000–900 cm^−1^ at a spectral resolution of 1 cm^−1^ maintained at 22 °C. A dry air stream was continuously circulated throughout the measurement process via an air compressor sourced from Jun-Air (Munich, Germany). Background readings (buffer solution) were collected following identical procedures. Data processing and interpretation were accomplished using the Opus 7.0 software package.

Principal component analysis (PCA) was performed to evaluate the relationships between the FTIR spectral descriptors and identify the main sources of structural variation among liposomal formulations, as described elsewhere [22]. The analysis was based on the shifts in the asymmetric methylene stretching band (Δν_as_CH_2_), carbonyl band (ΔνC=O), and phosphate band (ΔνPO_2_^−^) that were induced by linezolid incorporation relative to the corresponding blank liposomes. Prior to analysis, all variables were standardized using Z-score normalization. PCA was carried out using the scikit-learn package (Python 3.12.13). The score plots and loading plots were used to visualize clustering of formulations and assess the contribution of individual spectral descriptors to the principal components.

### 2.10. The Dialysis-Based Linezolid Release from Liposomal Formulations

The dialysis-based linezolid release from the liposomal formulations was evaluated using the dialysis method. Dialysis bags with a molecular weight cut-off of 12–14 kDa (Orange Scientific, Braine-l’Alleud, Belgium) were equilibrated in the corresponding release medium before use. A solution of non-encapsulated linezolid at the same initial drug concentration as that in the liposomal formulations was used as a control and examined under identical conditions.

Two release media were used: a sodium phosphate buffer solution 0.01 M, pH 7.4 (BS), and the same solution containing 1% (*w*/*v*) bovine serum albumin (BSA). For the experiments in the protein-containing medium, a 10% (*w*/*v*) stock BSA solution was added directly to each liposomal dispersion to obtain a final BSA concentration of 1% (*w*/*v*) before loading the sample into the dialysis bag. The external release medium contained BSA at the same concentration. For the experiments performed without BSA, an equal volume of BSA-free buffer was added to the liposomal dispersions to maintain identical sample volumes and dilution under both experimental conditions.

An aliquot of 0.75 mL of each liposomal formulation was placed into a dialysis bag and immersed in 10 mL of the corresponding release medium. The experiments were conducted at 37 °C under continuous stirring at 120 rpm for 24 h. At predetermined time points, 1.0 mL aliquots were withdrawn from the external medium and immediately replaced with an equal volume of fresh release medium maintained at 37 °C. All experiments were performed using three independent liposome preparations.

The linezolid concentration in the external medium was determined by UV spectrophotometry at 250 nm using calibration curves prepared in the corresponding release medium. For measurements performed in the presence of BSA, the same BSA-containing medium without linezolid was used as the spectroscopic blank. The background absorbance of the corresponding release medium was subtracted from each measurement, and the amount of linezolid transferred into the external compartment was calculated using the corresponding calibration equation. The results are reported as apparent cumulative linezolid release under the selected dialysis conditions.

To account for the periodic sampling and replacement of the release medium, the cumulative amount of released drug (*Q_n_*) was calculated according to Equation (3) as follows:

(3) $$Q_{n}=C_{n}\times V_{0}+V_{s}\times \sum C_{i\;}\;\;$$

where *C_n_* is the concentration of linezolid in the release medium at sampling point n, *V_0_* is the total volume of the release medium (10 mL), *V_s_* is the withdrawn sample volume (1 mL), and *C_i_* represents the concentrations measured at all previous sampling points.

The cumulative release percentage was calculated according to Equation (4):

(4) $$Release\;\left(\%\right)=\frac{Q_{n}}{M_{0}}\times 100\%\;\;$$

where *M_0_* is the initial amount of linezolid loaded into the dialysis bag.

Drug release models were considered as following. The zero-order release model is described by Equation (5):

(5) $$Q_{t}\;={\;Q}_{\infty }\;+\;K_{0}t,$$

where $K_{0}$ is the zero-order release constant, min^−1^; $Q_{t}$ is the amount of substance released at time t; and $Q_{\infty }$ is the total amount of substance released.

The first-order release model corresponds to Equation (6):

(6) $$Q_{t}\;=\;Q_{\infty }\;\times \;e^{-K_{1}t},$$

where $K_{1}$ is the first-order release constant, min^−1^.

The Korsmeyer–Peppas release is described by Equation (7):

(7) $$\frac{Q_{t}}{Q_{\infty }}\;=\;K\;\times \;t^{n},$$

where n is the release index; and K is the degree of release constant, min^−1^.

The Higuchi release model is represented by Equation (8):

(8) $$Q_{t}\;=\;A\;\times \;\sqrt{\frac{D\;\times \;\varepsilon }{\tau }\;\times \;\left(2\;\times \;C-\varepsilon \;\times \;C_{s}\right)\;\times \;C_{s}\;\times \;t}\;=\;K_{H}\;\times \;\sqrt{t}\;,$$

where $K_{H}$ is Higuchi dissolution constant, min^−1/2^; A is the unit of surface from which the amount of substance $Q_{t}$ is released during time t; *D* is the diffusion coefficient, sm^2^/min; *C* is the initial concentration of the released substance; $C_{s}$ is the solubility of the substance in the matrix; ε is the porosity of the matrix (the number of channels and pores); and τ is the tortuosity factor of the capillary system of the matrix (the value of the radius and the branching of the channels and pores within the matrix).

### 2.11. Antibacterial Activity Tests In Vitro

Antibacterial activity studies using solid media (LB pH 7.4) were carried out via the agar diffusion method [23]. We used freshly prepared linezolid solution and two linezolid liposomal forms diluted in purified sodium phosphate buffer solution at pH 7.4 by a syringe filter with 200 nm pore size. As the negative control, the unloaded liposomes were investigated. An overnight *Bacillus subtilis* ATCC 6633 (All-Russian Collection of Industrial Microorganisms, Kurchatov Institute, Moscow, Russia) was diluted to a 0.5 McFarland turbidity standard. Then, 500 µL of the bacterial suspension was spread over the surface of the solid nutrient medium. After 15–20 min, agar disks with a diameter of 9 mm were removed by a sterile plastic tip. Each sample (50 µL) was placed in the agar well. Petri dishes were incubated at 37 °C for 24 h. The diameters of the bacterial growth inhibition zones were measured, and the total clear-zone area, including the area of the 9 mm agar well, was calculated as $S=\pi d^{2}/4$, where d  is the measured zone diameter. An agar diffusion-derived apparent minimum inhibitory concentration (MIC_app_) was estimated from the linear regression of the total inhibition-zone area against the decimal logarithm of the linezolid concentration, using the low-concentration range of 0.9–2.0 μg/mL. The regression equation was $S=118.00\times {log}_{10}C_{Lzd}+89.57\;$($R^{2}=0.935$). An operational threshold of $S=70\;{mm}^{2}$, corresponding to a zone diameter of approximately 9.4 mm and therefore to the absence of a clearly measurable inhibition zone beyond the edge of the agar well, was used to estimate MIC_app_. The substitution of this threshold into the regression equation yielded a value of 0.683 μg/mL, which was reported as approximately 0.7 μg/mL. This value represents an empirical agar diffusion-derived apparent MIC. The experiments were performed independently three times, and the results are presented as mean ± SD.

For antibacterial activity studies in liquid media (LB pH 7.4), the overnight culture was diluted twice with fresh media. Then, 1.8 mL of bacterial culture was mixed with 0.2 mL of the sample (C_Lzd in the sample_ = 0.7 μg/mL) in a sterile tube. The tubes were shaken at 150 rpm (37 °C) for 6 h. The optical density of the aliquots was measured at 600 nm. The experiments were carried out independently three times, and the obtained values are averaged and presented with a standard deviation.

### 2.12. Ex Vivo Tuberculosis Granuloma Model

The study was conducted in accordance with the Declaration of Helsinki. Written in-formed consent was obtained from the healthy tuberculin-positive donor before blood collection. The protocol No2/1 was approved 25 February 2026 by the Local Ethics Committee of the Federal State Institution National Medical Research Center of Tuberculosis and Infectious Diseases of the Ministry of Health.

The ex vivo tuberculosis granuloma model was established using peripheral blood mononuclear cells (PBMCs) obtained from a healthy tuberculin-positive donor. Venous blood (15–20 mL) was diluted as 1:1 with phosphate-buffered saline, layered over Ficoll-Paque PREMIUM (GE Healthcare, Marlborough, MA, USA), and centrifuged at 2000× *g* for 20 min. The interphase layer containing mononuclear cells was collected and washed twice: first with physiological saline (30 mL), and then with RPMI 1640 medium without supplements (20 mL). Centrifugation was performed at 2000× *g* for 15 min. The supernatant was discarded, and the pellet was resuspended in RPMI 1640. Cell counting was performed using an automatic Scepter 2.0 cell counter (Sigma-Aldrich, St. Louis, MI, USA). The PBMC suspension was adjusted to a density of at least 5.0 × 10^5^ cells/mL and seeded into 24-well plates at 500 µL per well. Then, 500 µL of complete RPMI 1640 medium, supplemented with 10% fetal bovine serum (FBS), L-glutamine, penicillin/streptomycin, non-essential amino acids, vitamins, and sodium pyruvate, was added to each well.

On the following day, *Mycobacterium tuberculosis* H37Rv culture, grown in Middlebrook 7H9 liquid medium (Himedia, Mumbai, India) to the exponential phase (fluorescence index of 400 growth units in the BACTEC MGIT system), was added at a mononuclear cell:mycobacteria ratio of (5–6):1. To determine the initial mycobacterial inoculum, serial ten-fold dilutions of the culture were plated onto Middlebrook 7H10 solid medium and analyzed by quantitative real-time PCR targeting the multi-copy IS6110 gene (FAM channel) and the single-copy regX3 gene (ROX channel). Figure S2 illustrates the maturation of granulomas.

Cultures were maintained at 37 °C in a 5% CO_2_ atmosphere. The medium was replaced every 48 h. Granuloma formation was monitored daily using an inverted Leica DMIL LED microscope(Leica Microsystems GmbH, Wetzlar, Germany) (×200 magnification, Leica DFC 420 camera). Well-formed granulomas were typically observed on days 13–15 of incubation (Figure S2). The criterion for successful granuloma formation was a mononuclear cell:mycobacteria ratio of at least 5:1, accompanied by the presence of characteristic cellular aggregates that were visualized microscopically.

After granuloma formation, the tested liposomal linezolid formulations were added to the wells twice (at a 24 h interval) at 50 µL per well. Forty-eight hours after the second addition, the contents of the wells were collected, serially diluted, and plated onto Middlebrook 7H10 agar plates. Colony-forming units (CFU) were counted after 21 days of incubation at 37 °C.

All granuloma experiments were performed using PBMCs obtained from a single healthy tuberculin-positive donor. The three wells per formulation represent technical replicates and not independent biological replicates. Therefore, the results are presented as exploratory single-donor evidence.

### 2.13. Statistical Analysis

All experiments were performed in at least triplicate, and the results are presented as mean ± SD. Unless otherwise specified, *n* = 3 denotes three independently prepared liposome batches produced in separate experimental runs starting from lipid-film preparation. Multiple instrumental readings or video acquisitions obtained from the same batch were treated as technical acquisitions and were averaged to provide one value per independent preparation; they were not treated as independent observations in the statistical analysis. The PBMC-derived granuloma experiment was the only exception and consisted of three technical replicate wells obtained using PBMCs from a single donor. These data were analyzed descriptively without inferential statistical testing.

Statistical analyses were performed using AtteStat 3.04 for Microsoft Excel and Python 3.13 (SciPy and statsmodels packages). The significance of differences between two independent groups was assessed using the Mann–Whitney U-test, with *p* ≤ 0.05 considered statistically significant. The effects of the cholesterol content, drug-to-lipid ratio, and release medium on dialysis-based linezolid release/transfer were evaluated using two-way or three-way analysis of variance (ANOVA), as appropriate. Prior to ANOVA, the assumptions of normality and homogeneity of variances were assessed using the Shapiro–Wilk and Levene tests, respectively. Partial eta-squared (ηp^2^) was calculated as a measure of effect size. Statistical significance was accepted at *p* < 0.05.

## 3. Results

### 3.1. Liposomal Form of Linezolid Preparation

Lzd-loaded liposomes were prepared by the thin-film hydration method followed by ultrasonication. Soy lecithin and cholesterol were used as the matrix lipids, with the cholesterol content varied at 10, 20, and 30 wt% relative to the total lipid mass. Three drug-to-lipid loading levels were examined—S (0.1 mg Lzd per 10 mg lipids, 1:100 *w*/*w*), M (0.1 mg per 5 mg lipids, 1:50 *w*/*w*), and L (0.1 mg per 2 mg lipids, 1:20 *w*/*w*)—yielding nine distinct formulations. The encapsulation efficiency of Lzd, determined by dialysis followed by UV spectrophotometry, was ≥95% for all formulations, indicating near-quantitative association of the drug with the liposomal bilayer regardless of the cholesterol content or drug loading level. As a control, we considered 100% soy lecithin formulations. The freshly prepared vesicles 0-M were of the same range of parameters as those containing cholesterol. However, their diameter increased markedly during storage, exceeding 450 nm after 2 days of storage and 600 nm after 3 days while being accompanied by visible turbidity. In contrast, unloaded cholesterol-free liposomes prepared using the same procedure remained colloidally stable for at least two weeks. This difference indicates that the rapid destabilization was associated with the combined effects of linezolid loading and the absence of cholesterol rather than with sonication alone.

The cumulant-derived DLS Z-average diameters of the formulations ranged from 73 to 148 nm, with all samples exhibiting PDI values below 0.20 (Table 1). NTA yielded arithmetic mean hydrodynamic diameters of 74–105 nm from the number-based distributions of optically tracked particles. Because the DLS Z-average and the NTA arithmetic mean represent different measurands, their numerical values were interpreted as method-specific, complementary descriptors rather than as directly interchangeable estimates of the same average particle diameter.

For several formulations, the NTA-derived arithmetic means exceeded the corresponding DLS Z-averages. This direction of discrepancy is not unprecedented: NTA-derived mean diameters exceeding DLS Z-average values have previously been reported for PLGA nanoparticles, liposomes, and other polydisperse vesicle systems [24,25,26]. Representative method-specific particle-size distributions obtained for the formulation 10-M are shown in Figure S3. No visible sedimentation or precipitation was observed before or during the measurements.

Neither the cholesterol content nor Lzd loading alone produced a monotonic effect on vesicle size. Instead, the data revealed a pronounced interaction between these two formulation variables. According to the DLS Z-average values, the smallest vesicles were obtained for the formulation 20-M, whereas low-loaded formulations containing 20 or 30 wt% cholesterol exhibited larger Z-average diameters. The ζ-potential of all formulations remained negative, ranging from –16.9 to –43.3 mV (Table 1). The effect of Lzd loading on the vesicles’ surface charge strongly depended on the cholesterol content. At 10 and 20 wt% cholesterol, increasing the drug loading generally reduced the absolute ζ-potential, whereas at 30 wt% cholesterol, an opposite tendency was observed, with low and high loading resulting in more negative ζ-potentials than those of the corresponding unloaded liposomes.

Although cholesterol is electrically neutral, the formulations containing 30 wt% cholesterol exhibited more negative ζ-potential values. As Chol is neutral, this phenomenon could not be caused by the introduction of additional negative charges in the membrane. Instead, cholesterol can modify the phospholipid headgroup packing, interfacial hydration, and association of ions with the membrane surface. Magarkar et al. [27] showed that increasing the cholesterol content in zwitterionic DSPC and POPC membranes reduced Na^+^ binding to the phospholipid headgroups and shifted the ζ-potential toward more negative values. Increased absolute ζ-potential values at higher cholesterol contents have also been reported for lecithin- and phosphatidylcholine-based liposomes [28,29]. Thus, the present results are consistent with cholesterol-dependent reorganization of the membrane–solution interface.

Taken together, these results indicate that the colloidal properties of the liposomes are governed by the combined influence of cholesterol content and Lzd loading rather than by either factor alone. The non-monotonic behavior observed for both particle size and ζ-potential suggest that Lzd’s incorporation induces cholesterol-dependent changes in bilayer organization. To gain molecular-level insight into these effects, the lipid membrane was further investigated by ATR-FTIR spectroscopy.

### 3.2. ATR-FTIR Studies Reveal Main Structural Differences Between Liposomal Forms of Linezolid Depending on DLR and Lipid Composition

To obtain detailed information on the state of individual functional groups within the bilayer, we employed the well-established method of attenuated total reflection Fourier-transform infrared (ATR-FTIR) spectroscopy. This approach allowed us to examine the molecular organization of the lipid bilayer in liposomes comprising soy lecithin and cholesterol, as well as the effect of Lzd incorporation at various lecithin-to-cholesterol ratios and different drug loading levels. We analyzed the positions of four main characteristic absorption bands sensitive to structural rearrangements in the membrane: the asymmetric and symmetric stretching vibrations of CH_2_ groups in the acyl chains, reflecting the mobility of the hydrophobic tails; the stretching vibration of the carbonyl group, located at the lipid–water interface and sensitive to changes in hydration; and the asymmetric stretching vibration of the phosphate group, serving as a marker for electrostatic interactions and hydration in the headgroup region. Table 2 presents the positions of these bands for the liposomes under investigation.

We first examined the effect of cholesterol on the structure of unloaded liposomes (Table 2). Liposomes solely comprising soy lecithin exhibited spectral characteristics typical of a hydrated liquid–crystalline bilayer.

Incorporation of 10 wt% cholesterol induced only minor spectral changes in both the hydrophobic and interfacial regions of the membrane. The ν_as_CH_2_ band shifted by 0.9 cm^−1^ to higher wavenumbers, while only small changes were observed in the carbonyl and phosphate regions, indicating limited perturbation of membrane organization.

More pronounced effects emerged upon increasing the cholesterol content to 20 wt%. While the ν_as_CH_2_ band remained essentially unchanged, the carbonyl stretching band shifted from 1736.4 to 1733.2 cm^−1^, indicating increased hydration of the carbonyl groups, which has also been demonstrated in [30]. This observation suggests that cholesterol primarily affects the interfacial region of the bilayer rather than the acyl-chain region within this concentration range. Similar behavior has been reported previously and attributed to reorganization of the interfacial hydrogen-bonding network.

A qualitatively different spectral pattern was observed at 30 wt% cholesterol. In addition to a modest high-frequency shift in the ν_as_CH_2_ band, both the carbonyl and phosphate bands exhibited pronounced high-frequency shifts from 1733.2 to 1739.3 cm^−1^ and from 1216.9 to 1221.1 cm^−1^, respectively. The simultaneous dehydration of both carbonyl and phosphate groups indicates substantial reorganization of the membrane interface and a marked reduction in water accessibility within the headgroup region. Such behavior is consistent with previous reports describing cholesterol-enriched membrane domains in cholesterol-rich bilayers [31].

Notably, the most significant cholesterol-induced changes were observed for the carbonyl and phosphate groups rather than for the acyl-chain region. Thus, increasing the cholesterol content from 20 to 30 wt% appears to trigger a transition from a relatively hydrated membrane interface to a substantially dehydrated interfacial state. The observed phenomenon suggests the hypothesis that drug–membrane interactions are strongly influenced by the organization of the bilayer interface.

The effect of Lzd on the lipid bilayer was studied at three loading levels of S (low), M (medium), and L (high), corresponding to lipid-to-Lzd mass ratios of 100:1, 50:1, and 20:1, respectively. The incorporation of Lzd into the cholesterol-free formulation produced a small shift in the ν_as_CH_2_ band from 2926.0 to 2925.0 cm^−1^. The carbonyl band shifted from 1735.1 to 1733.0 cm^−1^, while the ν_as_PO_2_^−^ band shifted from 1217.1 to 1219.6 cm^−1^. Importantly, the absence of major spectral abnormalities in the freshly prepared cholesterol-free samples indicates that their subsequent colloidal destabilization was not caused by an immediate failure of bilayer formation. Instead, cholesterol was required to maintain the stability of the initially formed soy lecithin vesicles over time.

Table 2 lists the positions of the main bands for all formulations, and Figure S4 represents the radar (spider) plots constructed for each series. Shifts in these bands reliably indicate changes in the microenvironments of the corresponding functional groups of lipids [32,33].

The incorporation of Lzd induced systematic changes in all three spectral regions under consideration, indicating that the drug affects both the hydrophobic core and interfacial region of the bilayer. In all formulations, the ν_as_CH_2_ band shifted to lower wavenumbers by 0.9–2.8 cm^−1^, consistent with increased acyl-chain ordering and incorporation of linezolid into the membrane. The largest shift was observed for formulation 10-M (Δν = −2.8 cm^−1^), whereas the effect was less pronounced at higher cholesterol contents, suggesting that cholesterol partially attenuates the influence of linezolid on acyl-chain packing.

The carbonyl stretching band underwent a low-frequency shift in all Lzd-loaded formulations, indicating increased hydration of the carbonyl region. The effect was particularly pronounced in liposomes containing 30 wt% cholesterol, where the carbonyl groups of unloaded liposomes were initially the most dehydrated. These data indicate that Lzd perturbs the organization of the membrane interface and increases water accessibility in the carbonyl region. At lower cholesterol contents, the same trend was observed, although the magnitude of the effect was smaller.

The most pronounced changes were observed for the phosphate groups. At 10 and 20 wt% cholesterol, Lzd induced high-frequency shifts in the ν_as_PO_2_^−^ band, corresponding to partial phosphate dehydration. The magnitude of the effect reached 5.6 cm^−1^ in the 10-M and 10-L formulations. In contrast, formulations containing 30 wt% cholesterol exhibited the opposite behavior. In this series, Lzd causes substantial low-frequency shifts in the phosphate band, reaching 7.1 cm^−1^ for the formulation 30-L, indicative of increased phosphate hydration. Thus, increasing cholesterol content from 20 to 30 wt% resulted not merely in a quantitative change in the phosphate response but in a complete inversion of its direction.

Notably, the strongest Lzd-induced effects were observed in the carbonyl and phosphate regions rather than in the acyl-chain region. This finding indicates that the membrane interface represents the primary site of Lzd-induced bilayer reorganization. The opposite behavior of the phosphate groups at a low- and high-cholesterol content further suggests that the mode of drug–membrane interaction is strongly dependent on the initial organization of the cholesterol-containing bilayer.

**Figure 1:**
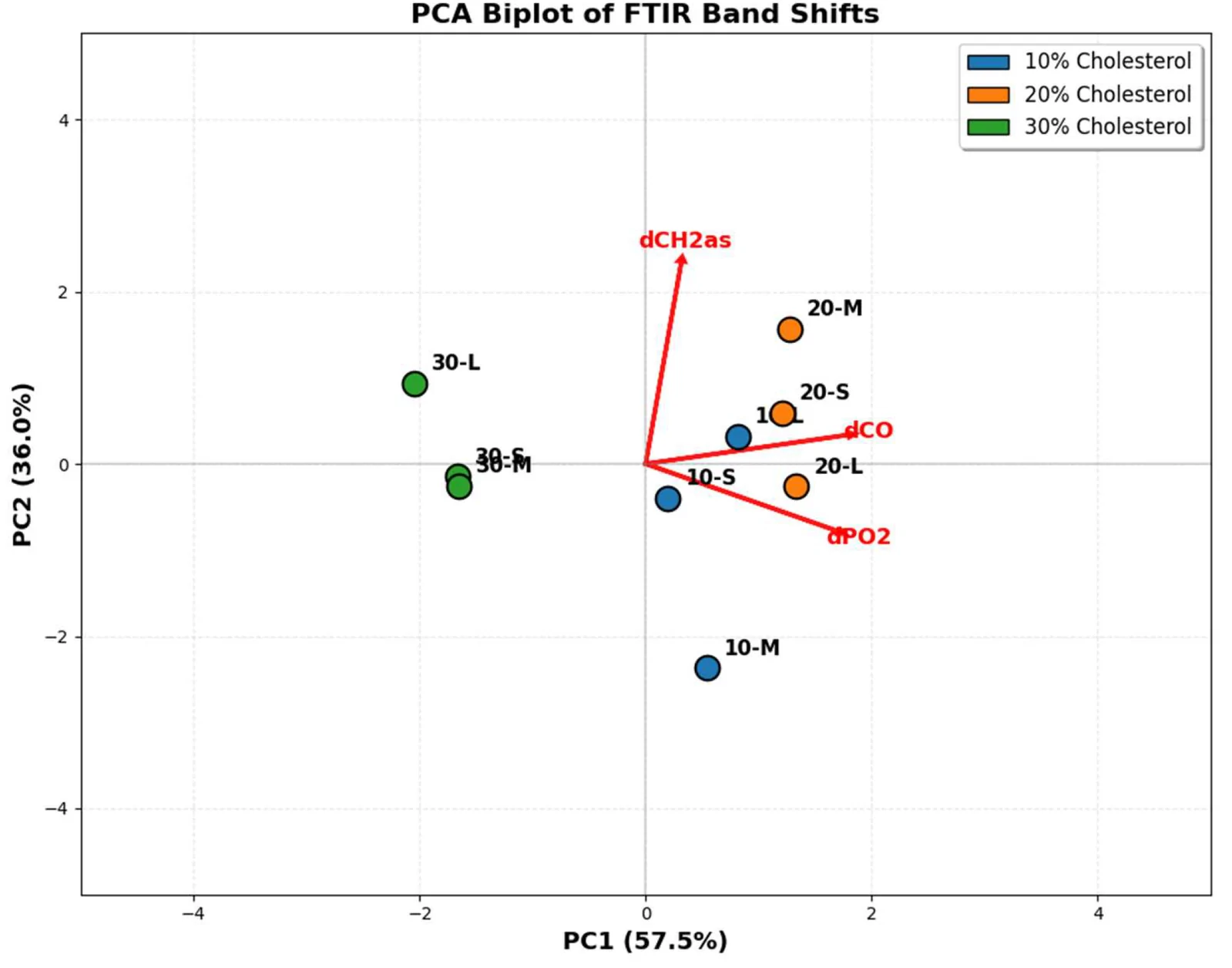
PCA biplot of FTIR spectral shifts induced by linezolid incorporation into liposomal bilayers. Analysis was performed using the changes in the ν_as_CH_2_, νC=O, and ν_as_PO_2_^−^ band positions relative to the corresponding unloaded liposomes. The first two principal components accounted for 93.5% of the total variance (PC1 = 57.5%, PC2 = 36.0%). PC1 was primarily associated with changes in the carbonyl and phosphate regions of the membrane interface, whereas PC2 was dominated by acyl-chain ordering reflected by the ν_as_CH_2_ band.

To facilitate the interpretation of FTIR data and identify relationships between spectral descriptors, principal component analysis (PCA) was performed using the Δν_as_CH_2_, ΔνC=O, and ΔνPO_2_^−^ values (Figure 1). The first two principal components explained 93.5% of the total variance (PC1: 57.5%; PC2: 36.0%). PC1 was mainly associated with the carbonyl and phosphate band shifts, whereas PC2 was dominated by the asymmetric CH_2_ stretching vibration. Thus, the multivariate analysis separated perturbations occurring in the membrane interface from those associated with acyl-chain ordering.

The plot revealed a clear separation of formulations according to cholesterol content, with formulations containing 30 wt% cholesterol occupying a distinct region of the PCA space. Notably, the separation was driven primarily by changes in the carbonyl and phosphate regions rather than by differences in acyl-chain ordering. These results indicate that interfacial reorganization and acyl-chain ordering represent partially independent modes of bilayer response to Lzd incorporation.

Together with the FTIR analysis, the PCA results support the conclusion that the membrane interface is the principal site of Lzd-induced structural reorganization, and that the nature of this response strongly depends on the cholesterol content of the bilayer.

### 3.3. Stability upon Storage and the Selection of Cryoprotectants for Freeze–Thaw Stability

**Figure 2:**
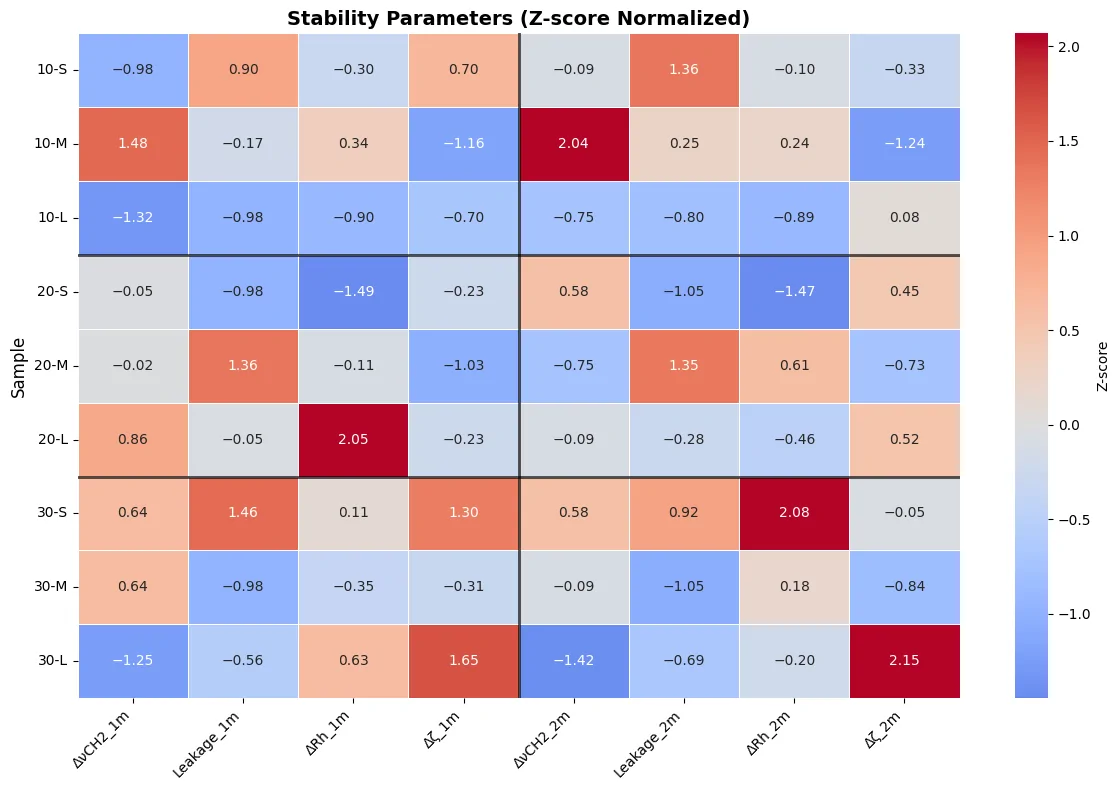
Heatmap summarizing storage-induced physicochemical changes in linezolid-loaded liposomes after one and two months of storage at 4 °C. Data were normalized as Z-scores for each parameter independently to facilitate comparison of relative changes across formulations. Blue and red colors indicate values below and above the mean of each parameter, respectively.

**Figure 3:**
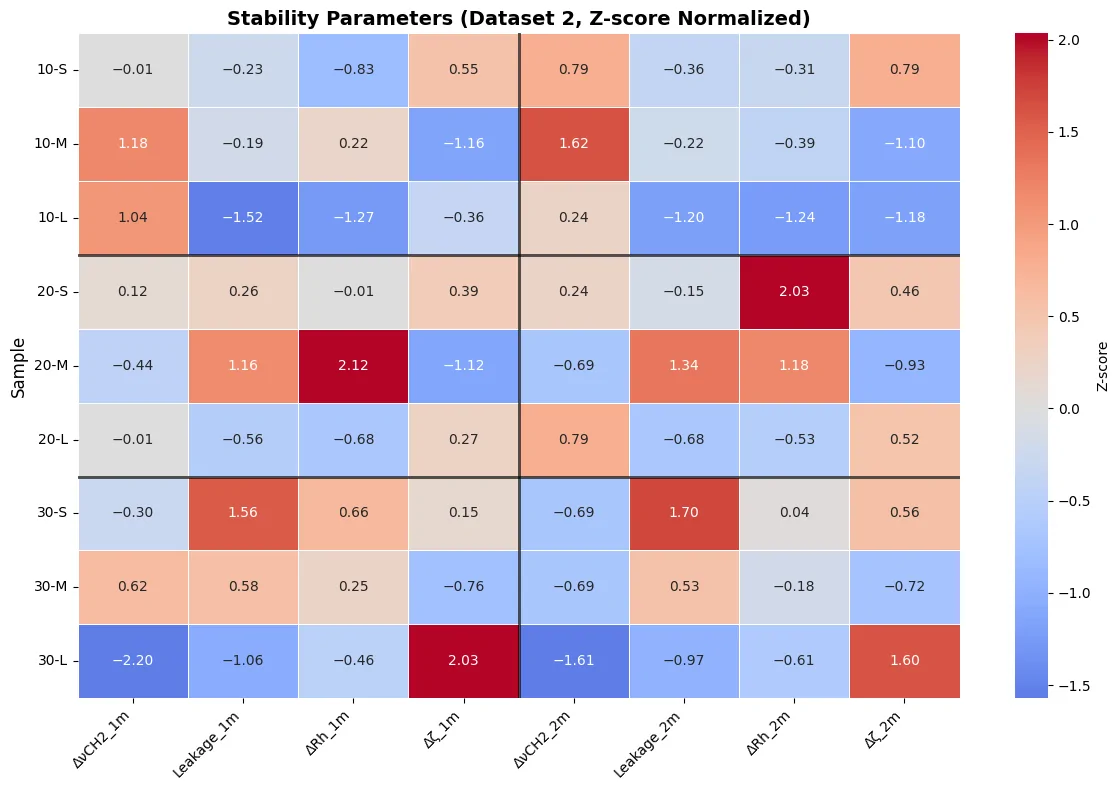
Heatmap summarizing storage-induced physicochemical changes in linezolid-loaded liposomes after one and two months of storage at −20 °C. Data were normalized as Z-scores for each parameter independently to facilitate comparison of relative changes across formulations. Blue and red colors indicate values below and above the mean of each parameter, respectively.

To evaluate the storage stability of the formulations, liposomes were stored at 4 °C and −20 °C for one and two months. Changes in membrane organization (ATR-FTIR), particle size, ζ-potential, and drug leakage were monitored. Because these parameters describe complementary aspects of liposome stability, the results were summarized as Z-score normalized heatmaps (Figure 2 and Figure 3), whereas complete numerical datasets are provided in the Supplementary Materials (Table S1).

Storage at 4 °C resulted in formulation-dependent changes without a common degradation pattern (Figure 2). Formulations differed in membrane rearrangement, particle-size variation, surface charge, and drug leakage, indicating that these descriptors reflect complementary rather than redundant aspects of stability. Among the investigated formulations, 10-L exhibited the smallest overall changes and the lowest drug leakage, whereas 30-S showed the largest deviations across several physicochemical parameters.

Frozen storage induced a characteristic two-stage response (Figure 3). After one month, the most pronounced changes were observed for the CH_2_ stretching band, indicating substantial perturbation of acyl-chain organization. After two months, these spectral changes became less pronounced, whereas alterations in particle size, ζ-potential, and drug leakage became more evident. This behavior suggests partial relaxation of the lipid bilayer following the initial freezing-induced structural perturbation.

Upon storage at −20 °C (Figure 3), a different pattern was observed. After one month, all formulations underwent a substantial high-frequency shift in the ν_as_CH_2_ band by 4–6 cm^−1^, indicating a pronounced disordering of the bilayer, most likely due to ice crystal formation and membrane damage during freezing and thawing. However, by the end of the second month, most samples recovered their initial ν_as_CH_2_ values within 0.5–1.0 cm^−1^, suggesting an ability of the bilayer to self-restore after thermal shock. The best stability upon freezing was demonstrated by the formulation 30-L, which showed the smallest shift after one month (only 0.5 cm^−1^) among all samples, and by two months the band returned to 2924.5 cm^−1^, nearly matching its initial value of 2926.9 cm^−1^.

While storage at 4 °C is suitable for short-term preservation, long-term stability often necessitates freezing, which can induce significant damage to liposomal vesicles. The primary mechanism of injury during freezing is the formation of ice crystals, which can exert mechanical stress on the lipid bilayer, leading to vesicle fusion, aggregation, and the leakage of encapsulated drugs [34,35]. To mitigate these effects, cryoprotective agents (CPAs) are employed. CPAs function by modulating ice formation and, more importantly, by forming a protective glassy matrix that stabilizes the vesicles during freeze–thaw cycles [34]. It is well-established that sugars and polyols, such as glucose and mannose, can serve as effective CPAs for liposomes by protecting the bilayer from the mechanical stress of ice crystals [35].

To identify suitable cryoprotectants for our LLzd formulation, we evaluated the protective effect of glucose and mannose. As a negative control, samples were subjected to rapid freezing by immersion in liquid nitrogen without any cryoprotectant. The integrity of the liposomes after freezing–thawing was assessed by monitoring changes in the hydrodynamic diameter (via DLS) and drug retention (via UV spectrophotometry).

Freezing at very low temperatures, such as when using liquid nitrogen (−196 °C), induced catastrophic damage to the unprotected liposomes. For the unprotected control, a dramatic increase in the hydrodynamic diameter was observed, indicating extensive vesicle aggregation and fusion. Concurrently, a substantial loss of the encapsulated Lzd was detected, confirming severe leakage. This outcome aligns with the literature’s reports, where the rapid formation of ice crystals during quenching in liquid nitrogen can strongly damage and fragment liposomes [35].

In contrast, the addition of glucose and mannose provided significant protection. Formulations containing these cryoprotectants mainly exhibited only minimal changes in their physicochemical properties post-thaw (Table S2). These results are consistent with previous studies demonstrating the cryoprotective efficacy of these sugars [34]. Although glucose has been reported to show weaker cryoprotective effects for some fluid lipid membranes [36], we found mannose to be suitable for our formulation based on soy lecithin and cholesterol.

The data clearly demonstrate that the inclusion of cryoprotectants is essential for the successful freezing of our liposomal formulations. Both glucose and mannose effectively preserved vesicle integrity and prevented drug leakage, whereas freezing without a cryoprotectant, particularly by rapid quenching in liquid nitrogen, resulted in irreversible damage. These findings underscore the importance of incorporating appropriate CPAs for the frozen storage of LLzd.

### 3.4. Dialysis-Based Linezolid Release

**Figure 4:**
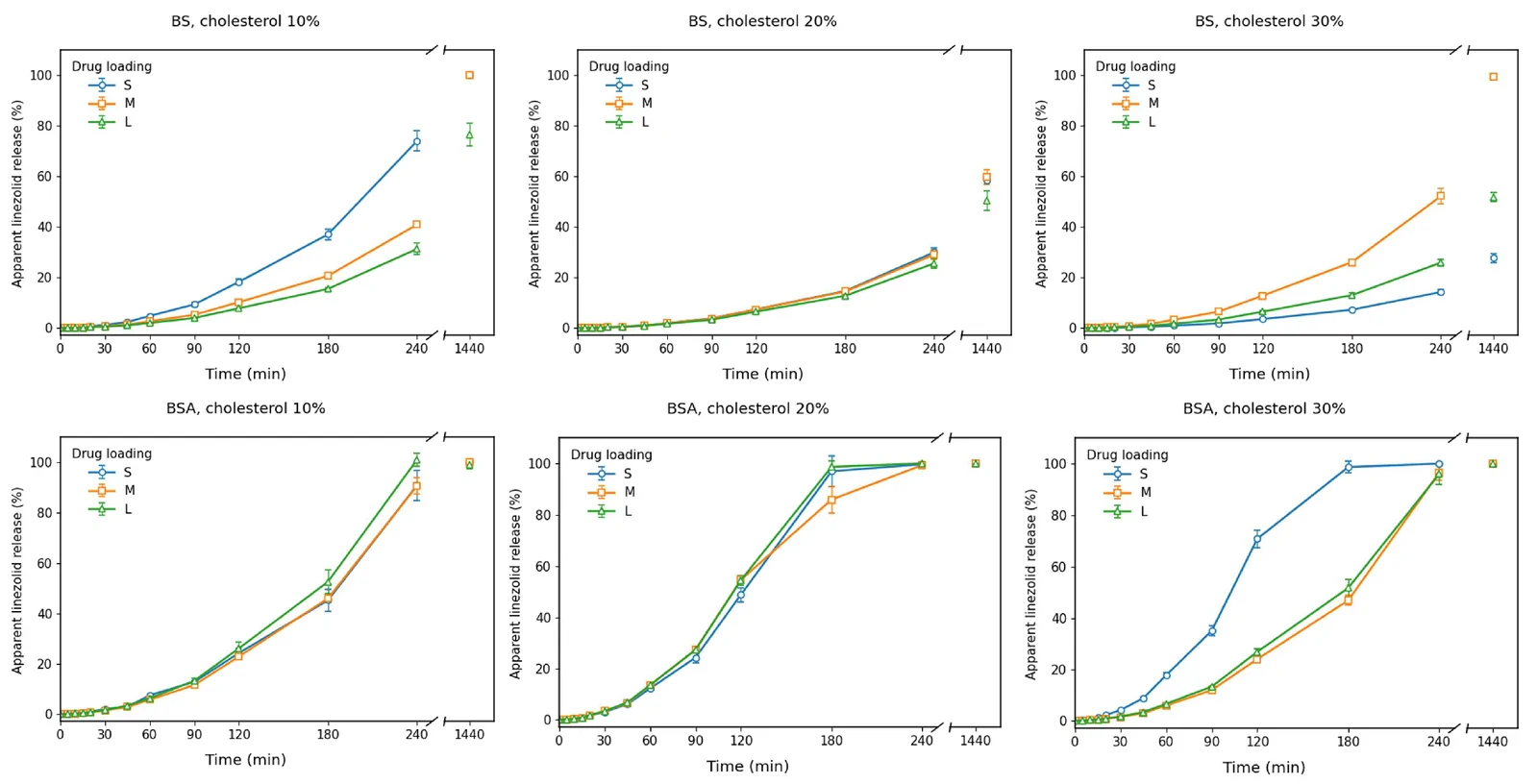
In vitro dialysis-based release profiles of linezolid from liposomal formulations in (BS) 0.01 M sodium phosphate buffer, pH 7.4, and (BSA) 0.01 M sodium phosphate buffer supplemented with 1% bovine serum albumin. Data are presented as mean ± SD from three independently prepared liposome batches (*n* = 3).

Figure 4 shows the dialysis-based release profiles of linezolid from liposomal formulations containing 10, 20, or 30 wt% cholesterol and three drug-to-lipid ratios (S, M, and L) in sodium phosphate buffer (BS, pH 7.4) and in the same medium supplemented with 1% bovine serum albumin (BSA). In this study, release was operationally defined as the cumulative transfer of linezolid from the liposomal dispersion to the acceptor compartment under the selected dialysis conditions. BSA was included to introduce protein-binding capacity and provide a more physiologically relevant release medium [37]. Albumin-containing dialysis media have previously been used to evaluate the drug release from liposomal and other nanocarrier formulations [37,38,39].

Although dialysis introduces an additional membrane-transfer step, free linezolid in BS crossed the membrane within approximately 20 min, whereas only a minor fraction of linezolid from the liposomal formulations was detected in the acceptor compartment during the first hour. The slower transfer of free linezolid in the presence of BSA (approximately 35 min) is consistent with reversible drug–albumin binding.

In BS, all formulations exhibited sustained release of linezolid over 24 h; however, the release profiles depended strongly on both the cholesterol content and drug loading (Figure 4 upper panel). At 10 wt% cholesterol, increasing the drug loading progressively reduced release at 240 min from 73.9 ± 4.1% (10-S) to 40.9 ± 0.6% (10-M) and to 31.2 ± 2.2% (10-L). In contrast, formulations containing 20 wt% cholesterol showed similar release values after 240 min (25.5–29.9%), whereas at 30 wt% cholesterol, a non-monotonic behavior was observed, with the formulation 30-M exhibiting a substantially higher release (52.1 ± 3.2%) than either 30-S (14.2 ± 1.0%) or 30-L (25.8 ± 1.2%).

The presence of 1% BSA markedly altered the release profiles (Figure 4, bottom panel). Most formulations exhibited substantially greater cumulative linezolid transfer than in BS, and the differences observed between formulations in the protein-free medium became considerably less pronounced. By 240 min, the fraction of linezolid that transferred to the acceptor compartment ranged from approximately 91% to 100% for all formulations. Thus, although BSA moderately delayed the dialysis-membrane transfer of non-encapsulated linezolid, it increased the overall transfer of linezolid from the liposomal dispersions.

To evaluate the combined effects of release medium, cholesterol content, and drug loading, the Lzd release at 240 min was analyzed using three-way ANOVA. Significant main effects were observed for the release medium (F = 7303.94, *p* < 0.0001), cholesterol content (F = 47.80, *p* < 0.0001), and drug loading (F = 20.21, *p* < 0.0001). Significant two-way interactions were also detected between the medium and cholesterol (F = 124.41, *p* < 0.0001), the medium and drug loading (F = 51.60, *p* < 0.0001), and cholesterol content and drug loading (F = 64.92, *p* < 0.0001). Importantly, a highly significant three-way interaction between the medium, cholesterol content, and drug loading was observed (F = 98.90, *p* < 0.0001, partial η^2^ = 0.917), indicating that the influence of formulation composition on dialysis-based linezolid release differed substantially between the BS- and BSA-containing medium.

The release profiles were additionally analyzed using several empirical kinetic models (Tables S3 and S4). Among them, the Korsmeyer–Peppas equation provided the best mathematical fit for the initial release stage (R^2^ = 0.982–0.999, Table S3), whereas the Higuchi model showed comparatively poor agreement with the experimental data. However, the high apparent transport exponents obtained (*n* from 1.75 to 2.14) indicate that these empirical models should not be interpreted mechanistically for the present liposomal system. Since the Korsmeyer–Peppas equation was originally developed for swellable polymeric matrices [40,41], such high *n* values are unlikely to represent classical Super Case-II transport in liposomal systems. Instead, they indicate that the observed dialysis-based profiles cannot be fully described by a simple diffusion-controlled power-law process. Because the measured profiles reflect a coupled process involving linezolid redistribution from the liposomal fraction, reversible binding, and transfer through the dialysis membrane, the individual contribution of each process cannot be resolved using empirical kinetic models alone.

### 3.5. Antibacterial Activity In Vitro

The antibacterial activity of Lzd and LLzd was evaluated against *Bacillus subtilis* ATCC 6633 using two complementary approaches: agar diffusion and liquid culture [42]. *B. subtilis* was used as a rapidly growing, linezolid-susceptible Gram-positive model for preliminary comparison of free and liposomal Lzd, whereas antitubercular activity was evaluated separately against *M. tuberculosis* H37Rv in the ex vivo granuloma model.

**Figure 5:**
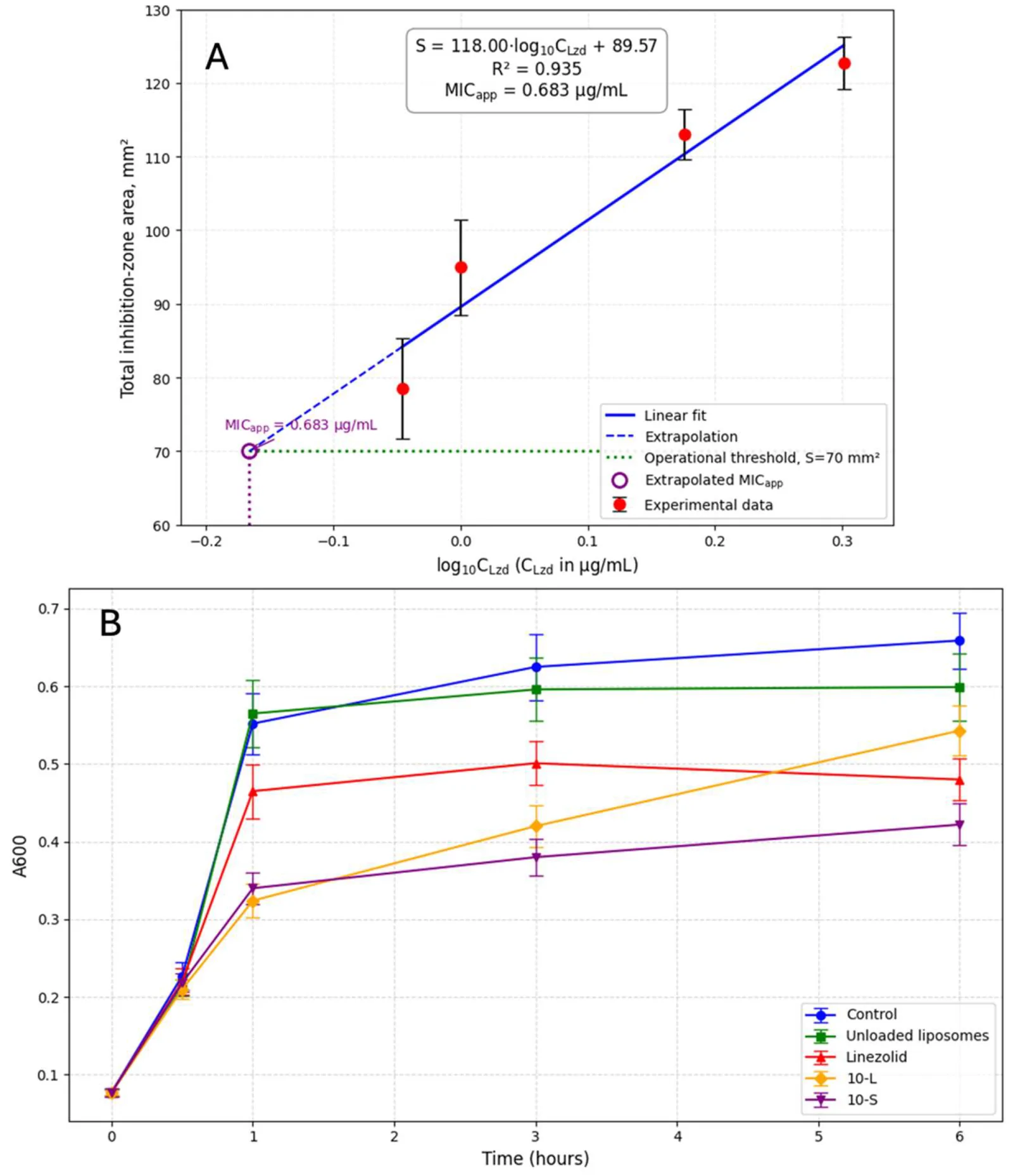
Antibacterial activity of free and liposomal linezolid against *Bacillus subtilis* ATCC 6633. (**A**) Estimation of the agar diffusion-derived apparent minimum inhibitory concentration (MIC_app_) of free linezolid. The solid line represents the linear regression, while the dashed segment represents extrapolation below the lowest experimentally tested concentration. MIC_app_ was operationally defined as the concentration corresponding to a predicted total inhibition-zone area of 70 mm^2^ and was estimated as 0.683 μg/mL. (**B**) Growth kinetics of untreated bacteria, bacteria incubated with unloaded liposomes, free linezolid, or linezolid-loaded formulations 10-L and 10-S in liquid LB medium, monitored by absorbance at 600 nm. C_Lzd in the sample_ = 0.7 μg/mL. Data are presented as mean ± SD from three independent experiments.

The agar well-diffusion assay was used as a rapid comparative method for evaluating the concentration-dependent antibacterial activity of free and liposomal Lzd. Free Lzd produced a dose-dependent increase in the bacterial growth inhibition zone. Linear regression of the total inhibition-zone area against log_10_C_Lzd_ in the low-concentration range of 0.9–2.0 μg/mL yielded an agar diffusion-derived apparent minimum inhibitory concentration (MIC_app_) of approximately 0.7 μg/mL (Figure 5A). At corresponding nominal linezolid concentrations, the tested liposomal formulations retained detectable antibacterial activity of a comparable magnitude, indicating that liposomal incorporation did not cause an obvious loss of detectable antibacterial activity in the agar diffusion assay.

Because inhibition-zone formation depends on the diffusion of both free Lzd and liposomal formulations through the agar matrix, MIC_app_ was interpreted as an assay-specific empirical threshold rather than as a standardized measure of intrinsic antibacterial potency. To further compare free and liposomal Lzd under conditions that allowed prolonged interaction with bacterial cells while avoiding diffusion through a gel matrix, the bacterial growth was subsequently monitored in a liquid culture (Figure 5B).

Both the untreated bacterial cultures and negative controls containing unloaded liposomes exhibited a rapid increase in optical density during the first hour of incubation, confirming normal bacterial proliferation and the absence of antibacterial activity of the lipid carrier itself. The addition of Lzd, either in a free or liposomal form, at the apparent MIC markedly suppressed bacterial growth. Liposomal formulations consistently produced slightly stronger growth inhibition than free Lzd, while the incorporation of cholesterol into the lipid bilayer resulted in a modest additional enhancement of the antibacterial effect. Although these differences remained relatively small, they suggest that liposomal encapsulation may improve the interaction of Lzd with bacterial cells under dynamic incubation conditions.

Although the liposomal formulation did not exhibit significantly enhanced antibacterial activity against bacteria compared with free linezolid, this observation is consistent with previous reports for a number of liposomal antibiotics [42,43]. Standard broth susceptibility assays do not fully capture the biological advantages of liposomal drug delivery, which are primarily associated with improved interaction with host cells and altered drug distribution within infected tissues. To investigate whether these advantages translate into enhanced efficacy under physiologically relevant conditions, the formulation was subsequently evaluated in an ex vivo granuloma model.

### 3.6. Antibacterial Activity Ex Vivo in Tuberculosis Granuloma

**Figure 6:**
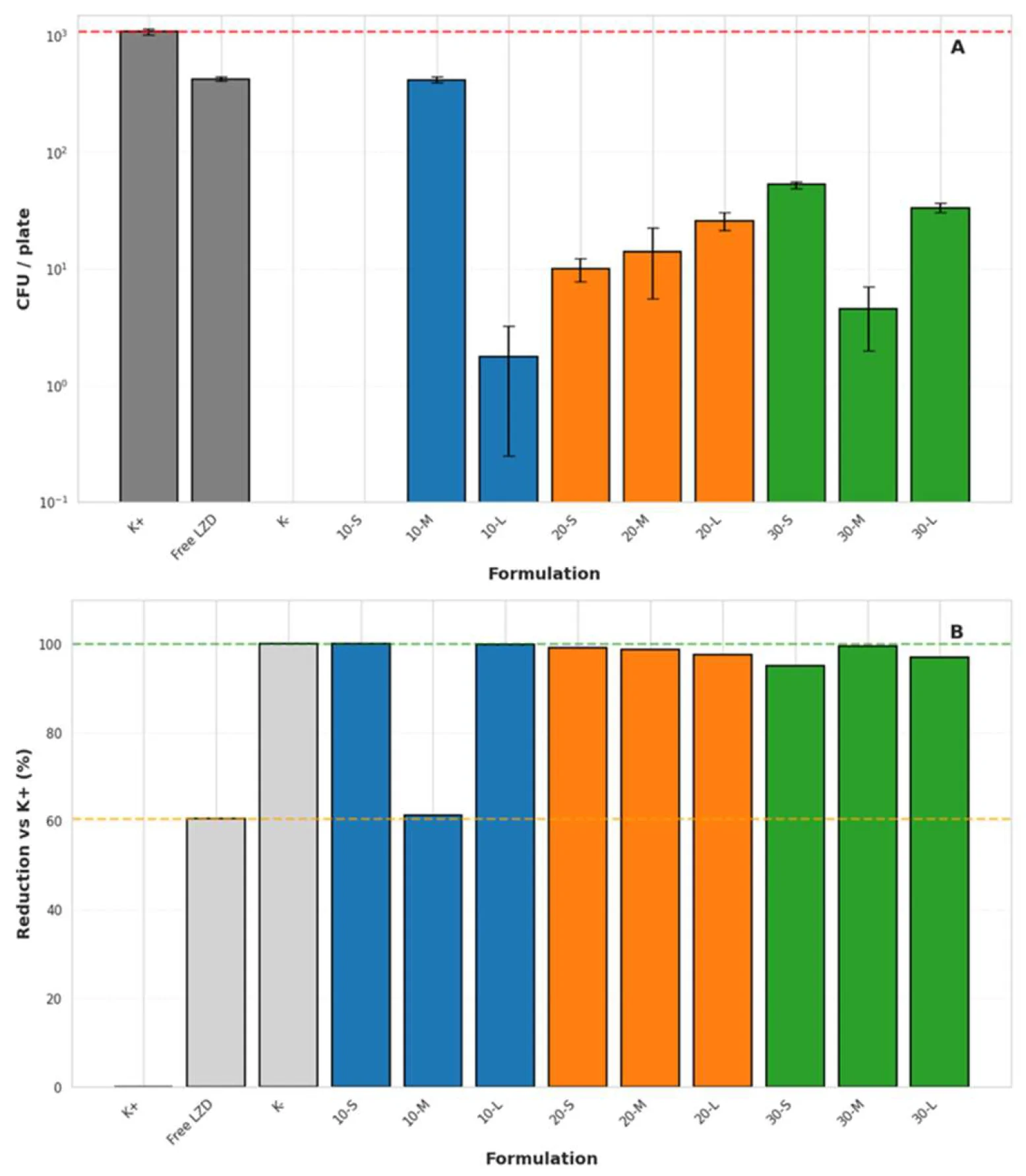
Antimycobacterial activities of liposomal linezolid formulations in the ex vivo tuberculosis granuloma model. (**A**) Bacterial load (CFU/plate) after treatment with free linezolid or liposomal formulations. Data are presented as mean ± SD from three technical replicate wells obtained using PBMCs from a single donor. K+—untreated infected control, K−—negative control. (**B**) Reduction in CFU relative to the untreated infected control (K+), calculated from the data shown in panel (**A**). The results are presented as exploratory single-donor evidence.

The antimycobacterial activity of free and liposomal Lzd formulations was evaluated using an ex vivo tuberculosis granuloma model (Figures S2 and Figure 6). The positive control exhibited a high bacterial burden (1075 ± 64.55 CFU/plate), confirming the viability of the inoculum and the suitability of the experimental model, whereas no bacterial growth was detected in the negative control.

Free Lzd, at a concentration of 0.1 mg/mL, reduced the bacterial burden to 423.75 ± 17.84 CFU/plate, corresponding to approximately 61% growth inhibition relative to the positive control. In contrast, all liposomal formulations demonstrated superior antimycobacterial activity when compared with the free drug (Figure 6A,B).

The strongest antibacterial effect was observed for the formulation 10-S, which completely suppressed bacterial growth (0 CFU/plate). Similarly high activity was observed for the formulations 10-L (1.75 ± 1.50 CFU/plate) and 30-M (4.50 ± 2.52 CFU/plate), corresponding to >99% inhibition of mycobacterial growth. Formulations 20-S and 20-M also showed pronounced activity, reducing the bacterial counts to 10 and 14 ± 8.49 CFU/plate, respectively.

A notable exception was formulation 10-M, which yielded 416 ± 23.59 CFU/plate, a value comparable to that of free Lzd. The remaining formulations (20-L, 30-S, and 30-L) demonstrated intermediate activity, with bacterial counts ranging from 25.75 ± 4.35 to 52.50 ± 3.54 CFU/plate.

Overall, the results indicate that the encapsulation of Lzd into liposomal carriers substantially enhances its antimycobacterial activity in the granuloma model. Importantly, the biological response was strongly dependent on liposomal composition, with the formulations 10-S, 10-L, and 30-M exhibiting near-complete suppression of *M. tuberculosis* growth.

## 4. Discussion

The comprehensive characterization of the nine liposomal formulations revealed that their physicochemical properties, storage stability, release behavior, and antibacterial performance cannot be explained by cholesterol content or drug loading alone. Instead, these characteristics are governed by cholesterol-dependent changes in the molecular organization of the lipid bilayer, particularly within the membrane interface. By combining ATR-FTIR spectroscopy with physicochemical characterization, release studies, and biological evaluations, we propose a mechanistic model that links membrane organization with functional performance and provides a rationale for the design of liposomal Lzd formulations for tuberculosis therapy.

### 4.1. Molecular Organization of the Bilayer: Insights from ATR-FTIR

ATR-FTIR spectroscopy demonstrated that the incorporation of Lzd affects both the hydrophobic region of the membrane and the lipid–water interface. The low-frequency shift in the ν_as_CH_2_ band observed in all formulations indicates increased ordering of the acyl chains following drug incorporation. This effect was most pronounced in the formulations containing 10 wt% cholesterol and gradually diminished with the increasing cholesterol content, suggesting that cholesterol-rich membranes are less susceptible to drug-induced perturbation of the hydrocarbon chains. Loading-dependent decreases in acyl-chain mobility have also been reported for levofloxacin-containing liposomes, although the magnitude of this effect depended on the drug-to-lipid ratio and membrane composition [19]. Since cholesterol itself is known to modify the acyl-chain order, the smaller additional shift at higher cholesterol contents may reflect a reduced incremental effect of Lzd on an already compositionally constrained bilayer rather than the formation of a fundamentally different membrane state [14,15].

More substantial composition-dependent differences were observed in the carbonyl and phosphate spectral regions. These bands are sensitive to hydrogen bonding and hydration at the lipid interface, although their positions may also be affected by lipid packing, headgroup orientation, and local electrostatic interactions [13,44]. The changes in the carbonyl region following Lzd incorporation were consistent with an increased contribution of hydrogen-bonded carbonyl groups. The phosphate response, however, depended on the cholesterol content. At 10 and 20 wt% cholesterol, the phosphate band shifted towards higher frequencies, indicating reduced hydration of the headgroup region, whereas the 30 wt% formulations showed a shift in the opposite direction. These results suggest that the local environment of the phospholipid headgroups responds differently to Lzd at the highest cholesterol content. This phenomenon represents the possible structural distinction between low- and high-cholesterol formulations and suggests that increasing cholesterol content fundamentally alters the organization of the membrane interface.

Such composition-dependent behavior is consistent with previous evidence that the influence of cholesterol on membrane packing, and interfacial water is strongly dependent on the host lipid matrix. For example, cholesterol-induced changes in chain organization and interfacial water alignment differed markedly among unsaturated DOPC, saturated DPPC, and sphingomyelin membranes [17].

PCA was consistent with the interpretation that the largest spectral differences among the formulations were associated with the membrane interface. Carbonyl- and phosphate-region variables contributed substantially to sample separation, and the formulations containing 30 wt% cholesterol formed a distinct cluster within the analyzed dataset. As PCA is an exploratory method, it indicates that the spectral response of the 30 wt% cholesterol formulations differed predominantly in the interfacial region rather than exclusively in acyl-chain ordering.

### 4.2. Membrane Organization Influences on the Colloidal Stability

The composition-dependent differences revealed by ATR-FTIR were accompanied by variations in vesicle size, ζ-potential, and storage behavior. It should be noted that despite all liposomes being prepared using identical sonication conditions, vesicle size may also depend on membrane mechanical properties. Cholesterol increases the rigidity of phosphatidylcholine bilayers and has previously been reported to yield larger sonicated vesicles at higher cholesterol contents [45]. Consequently, membrane composition may influence not only bilayer organization but also the efficiency of vesicle size reduction during sonication. Since vesicle size itself can affect membrane curvature, colloidal stability, and drug retention, these factors cannot be considered entirely independent in the present study.

The DLS and NTA measurements were regarded as complementary rather than directly interchangeable descriptions of the liposomal dispersions. DLS provides a cumulant-derived Z-average based on the intensity autocorrelation function, whereas NTA provides a number-based distribution of individually tracked particles; consequently, direct comparison requires that the specific measurand reported by each method be clearly defined [24,46,47]. Previous comparative studies have similarly emphasized the complementary character of DLS and NTA for polydisperse nanoparticle dispersions [24,48].

NTA-derived mean diameters exceeding the corresponding DLS Z-average values have previously been reported. Filipe et al. obtained NTA mean diameters of 322 and 154 nm for PLGA nanoparticles and liposomes, respectively, compared to DLS Z-average values of 308 and 117 nm [24]. Kim et al. [26] likewise reported larger NTA-derived mean sizes for several polydisperse vesicle samples. Thus, although this direction of discrepancy may be contrary to the general expectation based on the stronger contribution of larger particles to DLS intensity, it is not unique to the present formulations.

As Malvern Panalytical’s application notes [49], comparing the same liposome samples by multi-angle dynamic light scattering and NTA further showed that NTA resolved a small shoulder corresponding to liposomes slightly larger than the principal population, whereas this minor population was too sparse and too close in size to the main population to be separately resolved by multi-angle dynamic light scattering. This observation provides a possible explanation for the somewhat larger NTA-derived mean diameters obtained for some of the present formulations. However, the presence of an analogous minor population in our samples was not directly established; this interpretation therefore remains tentative and is not regarded as evidence of aggregation, sedimentation, or a distinct vesicle population.

The contrasting stability of unloaded and linezolid-loaded cholesterol-free liposomes suggests that the rapid aggregation of 0-M was associated with drug-induced perturbation of the lecithin bilayer. The incorporation of linezolid into a cholesterol-free membrane may disrupt local lipid packing and promote aggregation. Cholesterol-induced membrane condensation and lateral organization may provide alternative ordered or interfacial environments for bilayer-associated linezolid, thereby reducing local packing stress and improving colloidal stability. However, because the precise intramembrane localization of linezolid was not directly determined, this explanation remains hypothetical rather than conclusive.

Within each cholesterol series, the formulations with intermediate drug loading generally exhibited the smallest hydrodynamic diameters. This finding indicates that vesicle size depended on the combination of cholesterol content and Lzd loading. The corresponding ATR-FTIR changes nevertheless support the conclusion that Lzd incorporation was associated with composition-dependent differences in membrane organization.

The reorganization of the membrane interface was also reflected in the ζ-potential. At 10 and 20 wt% cholesterol, Lzd loading generally reduced the absolute surface charge, consistent with reduced hydration of the phosphate region observed by ATR-FTIR. Conversely, formulations containing 30 wt% cholesterol exhibited more negative ζ-potential. These changes correspond to the spectral pattern in the phosphate region and may reflect changes in the local environment and exposure of phospholipid headgroups.

Storage experiments further demonstrated that membrane organization determines long-term stability. As such, 10-L showed the highest stability during refrigerated storage, whereas 30-L showed the most favorable behavior under frozen-storage conditions as well as limited Lzd leakage and relatively minor changes in hydrodynamic diameter and ATR-FTIR spectra. The higher stability of this formulation coincides with the pronounced hydration of both the carbonyl and phosphate regions, suggesting that a highly hydrated membrane interface contributes to preserving bilayer integrity during storage.

The addition of glucose or mannose reduced the changes observed after freezing and thawing, consistent with the established cryoprotective effects of carbohydrates and polyols in liposomal formulations. Previous studies have shown that the extent of protection depends on lipid composition, cryoprotectant identity and concentration, and the freezing protocol [34]. While cryoprotective agents may reduce freeze-induced leakage and aggregation, at sufficiently high concentrations, they may also perturb membrane organization and permeability [50].

The relatively small changes observed for 30-L after freezing may therefore reflect a combination of its initial membrane organization, cholesterol content, drug loading, vesicle size, and interaction with the cryoprotectant. Overall, the results identify the interfacial spectral state as one of several factors associated with colloidal and storage stability.

### 4.3. Membrane Organization Is Associated with Dialysis-Based Linezolid Release in Protein-Containing Medium

The release experiments demonstrated that both the cholesterol content and drug loading influence influenced the dialysis-based release behavior of linezolid from liposomes. More importantly, the statistical analysis revealed that these effects cannot be considered independently. The significant medium × cholesterol × drug loading interaction observed by three-way ANOVA indicates that the influence of formulation composition differed substantially between protein-free buffer and the protein-containing medium. Thus, BSA did not simply increase linezolid release by the same magnitude for all formulations but altered the relationship between liposome composition and release behavior.

The composition-dependent release profiles obtained in protein-free buffer correlate with the ATR-FTIR results. Formulations containing 10 wt% cholesterol generally exhibited higher release and a lower apparent hydration of the phosphate region, whereas higher cholesterol contents were associated with lower release for several formulation series. A reduction in drug leakage with increasing cholesterol content has previously been reported for a range of liposomal systems, including combretastatin A4-, celecoxib-, and azithromycin-loaded vesicles [16,18,51]. However, published studies also show that the magnitude and direction of the cholesterol effect depend on lipid composition, drug properties, and drug localization within the vesicle [16,52].

The previous studies further demonstrate that drug association with lipid membranes may exhibit a non-monotonic dependence on cholesterol content and may be influenced by preferential localization within cholesterol-rich domains or at membrane–domain interfaces [53,54,55]. These findings support the interpretation that cholesterol-dependent drug retention cannot necessarily be predicted from cholesterol concentration alone.

Experiments shown that cholesterol content alone was insufficient to predict linezolid retention and that its effect depended on drug loading. Similar loading-dependent changes in liposomal leakage have been reported for other drugs; for example, increasing the drug-to-lipid ratio increased combretastatin A4 leakage despite otherwise identical lipid composition [56]. Therefore, the release data are consistent with a combined contribution of cholesterol content, drug loading, and the resulting physicochemical state of the formulation; however, the relative contribution of each factor cannot be resolved from the release experiment alone.

In the BSA-containing medium, the composition-dependent differences observed in protein-free buffer were less pronounced for several formulations, although the response remained formulation-specific. Linezolid is known to bind serum albumin, and direct interactions between linezolid and BSA have been demonstrated spectroscopically [57]. In the present control experiments, the transfer of non-encapsulated linezolid through the dialysis membrane required approximately 35 min in the presence of BSA, compared with approximately 20 min in protein-free buffer. This moderate delay is consistent with a reduction in the freely diffusible linezolid fraction due to reversible albumin binding.

Importantly, BSA produced the opposite net effect in experiments with liposomal linezolid, as despite delaying the membrane transfer of the non-encapsulated drug, it substantially increased the cumulative transfer of linezolid from the liposomal dispersions. Therefore, the release profiles in the BSA-containing medium cannot be explained solely by a reduction in the freely diffusible linezolid fraction available for passage through the dialysis membrane.

In a liposomal dispersion, the freely dissolved linezolid concentration in the donor compartment is continuously replenished through the redistribution of the drug from the lipid phase into the aqueous phase. Albumin binding, particularly in the larger acceptor compartment, can reduce the freely dissolved drug concentration outside the dialysis membrane and thereby maintain effective sink conditions that favor continued transfer of linezolid from the liposomes.

Albumin-assisted dialysis approaches have previously been used to evaluate drug release from liposomes and other nanocarriers. Hioki et al. [37] demonstrated that the addition of BSA increased doxorubicin release from liposomal formulations and that the magnitude of the effect depended on liposome composition. Gil et al. [38] developed an albumin-assisted dialysis method for assessing the release of hydrophobic drugs from nanocarriers, including liposomes. Akaki et al. likewise evaluated controlled drug release from liposomes using a dialysis system containing serum and BSA [39]. These studies support the use of albumin-containing media for comparative evaluation of liposomal drug release, although the effects of albumin remain dependent on the properties of both the drug and the carrier.

Direct interactions between albumin and phospholipid vesicles may also contribute to the observed formulation-dependent response. Previous studies have shown that albumin can associate with phospholipid membranes and, under some conditions, promote lipid extraction or leakage of encapsulated solutes; the extent of these effects depends strongly on lipid composition and membrane organization [58,59,60]. However, the present experiments did not directly assess albumin adsorption. No visible precipitation or turbidity attributable to linezolid aggregation was observed under the experimental conditions. However, because drug aggregation was not investigated using a dedicated analytical method, it cannot be neither be confirmed nor completely excluded and was not used as the basis for mechanistic interpretation.

Dialysis introduces an additional membrane-transfer step and may distort release profiles when drug transport through the dialysis membrane is slower than release from the carrier [61]. Nevertheless, dialysis-based methods can provide relevant comparative information on drug release from liposomes when membrane transport and drug partitioning are appropriately considered [62].

In the present study, however, non-encapsulated linezolid crossed the membrane within approximately 20 min in protein-free buffer and 35 min in the BSA-containing medium, whereas only a minor fraction of linezolid was detected from the liposomal formulations during the first hour and formulation-dependent release continued for several hours. Membrane transfer was therefore unlikely to be the principal rate-limiting step over most of the investigated time interval, although the events occurring during the earliest sampling period cannot be resolved independently.

Accordingly, the dialysis curves are interpreted as comparative release profiles reflecting a coupled process that includes redistribution of linezolid from the liposomal fraction, reversible albumin binding, and transfer through the dialysis membrane.

Overall, the significant interaction between the release medium, cholesterol content, and drug loading supports an association between formulation composition and the response of the liposomes to a protein-containing environment. The correspondence with the ATR-FTIR data suggests that differences in lipid-chain ordering and the interfacial environment may contribute to this behavior.

The release profiles were additionally analyzed using several empirical kinetic models. The Korsmeyer–Peppas equation provided the highest coefficients of determination for all formulations (R^2^ = 0.982–0.999). This model was originally developed to describe solute release from polymeric matrices [63,64], although it has also been applied empirically to liposomal release profiles [65]. The transport exponents obtained here (*n* = 1.75–2.14) lie outside the ranges conventionally used to classify diffusion-controlled release from standard polymer geometries. They should therefore not be assigned to a classical transport mechanism. The kinetic fits are consequently interpreted descriptively rather than as evidence of a specific molecular release mechanism.

### 4.4. The Antibacterial Activity of Liposomal Linezolid

The antibacterial studies demonstrated that the relationship between physicochemical properties and biological activity is dependent on the experimental model. The agar well-diffusion assay was used as a preliminary, semi-quantitative screening method to derive an assay-specific apparent minimum inhibitory concentration MIC_app_ and to select an appropriate concentration range for subsequent antibacterial experiments. Under the selected assay conditions, free and liposomal Lzd showed similar MIC_app_ values of approximately 0.7 μg/mL. This finding indicates that the incorporation of Lzd into the liposomal formulations did not result in an obvious loss of detectable antibacterial activity.

However, MIC_app_ obtained by agar diffusion should not be considered equivalent to a standardized MIC determined by broth or agar dilution. Inhibition-zone formation depends not only on antibacterial potency but also on diffusion through the agar matrix, which may differ substantially between the freely dissolved Lzd and liposome-associated drug. Therefore, the similarity of the estimated thresholds does not demonstrate equivalent intrinsic potency or identical drug availability. Rather, the agar diffusion results provided an empirical reference for selecting the concentration used in the subsequent liquid-culture experiment, in which free and liposomal Lzd could be compared without the additional limitation imposed by diffusion through agar.

In the *B. subtilis* liquid-culture assay, all liposomal formulations produced greater growth inhibition than free Lzd under the tested conditions, although the differences among the formulations were relatively modest. The formulation 10-S showed the strongest inhibition during most of the incubation period. Its comparatively rapid apparent release may have contributed to faster drug availability in this model.

A different pattern was observed in the ex vivo granuloma model. The formulations 10-S, 10-L, and 30-M produced the greatest reduction in *M. tuberculosis* growth despite showing substantially different apparent release profiles. This result indicates that release behavior measured under dialysis conditions alone was limited to predict antibacterial performance in the granuloma model. The highly active formulations differed in ATR-FTIR spectral pattern, and thus, spectroscopic data could be considered an important part of the rationale design.

Overall, the results support the use of complementary antibacterial models with increasing biological complexity. The agar diffusion assay was useful for preliminary concentration selection, whereas the liquid-culture and granuloma experiments provided comparative information on formulation performance under different conditions. Antibacterial performance appears to arise from an optimal balance between membrane organization, protein responsiveness, and sustained drug availability, emphasizing the importance of evaluating liposomal formulations in biologically relevant models in addition to conventional bacterial cultures.

### 4.5. Proposed Model of Linezolid–Liposome Interaction

The combined experimental results support a composition-dependent structure–property–function relationship in linezolid-loaded liposomes. Cholesterol content and drug loading jointly affected the molecular organization of the membrane, as demonstrated by systematic changes in the acyl-chain, carbonyl, and phosphate regions of the ATR-FTIR spectra and by the distinct separation of the formulations in PCA. These structural differences were accompanied by corresponding changes in colloidal stability, drug retention, apparent release, and antibacterial performance. Thus, the effect of cholesterol cannot be reduced to a simple increase in membrane rigidity; instead, cholesterol modifies the way in which Lzd incorporation is accommodated within the lipid bilayer and at the lipid–water interface.

At 10 wt% cholesterol, Lzd incorporation produced the most pronounced increase in acyl-chain ordering and was accompanied by reduced apparent hydration of the phosphate region. These formulations generally exhibited higher apparent release in protein-free buffer and a stronger change in release behavior in the presence of BSA. Taken together, these findings support the existence of a comparatively responsive interfacial state in low-cholesterol membranes, in which the functional properties remain highly sensitive to drug loading and the surrounding medium. This behavior was particularly evident for 10-S, which combined rapid apparent release with strong antibacterial activity in the bacterial culture assay and in the ex vivo granuloma model.

Increasing the cholesterol content to 30 wt% produced a distinct spectral pattern, characterized by different responses of the carbonyl and phosphate regions and by separate clustering in the PCA score plot. These findings indicate a qualitatively different organization of the membrane interface at the highest cholesterol content. However, the functional consequences of this interfacial state remained dependent on drug loading. The formulation 30-L exhibited high storage stability and limited drug leakage, whereas 30-M showed substantially higher apparent release and strong antimycobacterial activity. Therefore, the 30 wt% cholesterol formulations behavior reflects the interaction between a cholesterol-rich membrane environment and the amount of incorporated Lzd.

The biological results further demonstrate that different combinations of membrane organization and release behavior can lead to higher antibacterial performance. The formulation 10-S achieved strong activity together with relatively rapid drug release, whereas 30-M combined a distinct high-cholesterol interfacial organization with a different release profile but similarly pronounced activity in the granuloma model. Antibacterial activity emerges from the combined effects of membrane organization, drug retention and availability, and the response of the formulation to the biological environment.

Overall, the present data support the structure–property–function framework. The cholesterol content and drug loading determine the physicochemical state of the liposomal membrane; this state, together with vesicle size and the surrounding medium, then shapes the storage stability, drug retention, and apparent release, and these properties collectively influence antibacterial performance. The framework is supported by the convergence of ATR-FTIR, PCA, colloidal stability, factorial release analysis, and biological data. At the same time, direct measurements of protein adsorption, macrophage uptake, intragranuloma distribution, intracellular Lzd concentration, and phagolysosomal delivery will be required to resolve the individual biological mechanisms underlying the observed formulation-dependent activity.

## 5. Conclusions

In this work, we systematically investigated the influence of cholesterol contents and drug-to-lipid ratios on the physicochemical properties, membrane organization, release behavior, storage stability, and antibacterial activity of linezolid-loaded liposomes. By integrating ATR-FTIR spectroscopy with colloidal characterization, release studies, and biological evaluation, we demonstrate that membrane organization, rather than individual formulation parameters, governs the functional performance of liposomal linezolid.

ATR-FTIR spectroscopy showed that Lzd incorporation affected both acyl-chain organization and the lipid–water interface, while the magnitude and direction of these changes depended on the cholesterol content. Formulations containing 30 wt% cholesterol exhibited a distinct interfacial spectral pattern, particularly in the carbonyl and phosphate regions. However, the functional consequences remained dependent on drug loading. Thus, cholesterol-rich formulations did not behave as a uniformly rigid or impermeable group.

Release analysis further demonstrated significant interactions between cholesterol content, drug loading, and release medium. The presence of BSA altered the composition-dependent release patterns rather than producing a uniform effect across all formulations. These findings indicate that neither cholesterol concentration, vesicle size, nor release rate alone is sufficient to predict formulation performance. Antibacterial activity was likewise model-dependent, with different physicochemical profiles resulting in strong activity in bacterial culture and in the ex vivo granuloma model.

Among the investigated formulations, 10-L provided the most balanced combination of storage stability, controlled release and antimycobacterial activity, making it the most promising candidate for further preclinical evaluation.

Overall, membrane interfacial organization emerged as an important integrative characteristic linking formulation composition with stability, drug retention, and biological performance. Further route-relevant safety, pharmacokinetic, biodistribution, and in vivo efficacy studies are required before the therapeutic potential of the formulations can be established. This concept may provide a general framework for the rational design of liposomal antibiotic delivery systems.

## Figures and Tables

**Table 1:** Physicochemical properties of liposomal linezolid formulations. Z-average radii, PDIs, NTA-derived arithmetic mean diameters, and ζ-potential values are presented as mean ± SD from three independently prepared liposome batches (*n* = 3). For NTA, five technical video acquisitions were obtained for each independent batch and averaged before calculation of the between-batch mean and SD. S, M, and L correspond to linezolid-to-lipid mass ratios of 1:100, 1:50, and 1:20, respectively. Measurements were performed in 0.01 M sodium phosphate buffer, pH 7.4, at 22 °C.

Sample	DLR, %	EE, %	Rh, nm (DLS)	PDI	Rh, nm (NTA)	ζ-Potential, mV
0 unloaded	-	-	100 ± 3	0.110	105 ± 3	−26.2 ± 1.4
0-M	2	96 ± 3	92 ± 5	0.124	98 ± 4	−25.4 ± 1.1
10 unloaded	-	-	101 ± 4	0.102	91 ± 3	−25.3 ± 0.9
10-S	1	96 ± 1	98 ± 3	0.135	90 ± 4	−26.6 ± 1.6
10-M	2	98 ± 1	76 ± 7	0.098	95 ± 3	−18.2 ± 2.7
10-L	5	97 ± 2	121 ± 4	0.112	96 ± 4	−16.9 ± 0.5
20 unloaded	-		112 ± 4	0.105	103 ± 2	−43.3 ± 1.2
20-S	1	95 ± 1	144 ± 4	0.127	98 ± 4	−31.3 ± 0.4
20-M	2	97 ± 2	73 ± 3	0.131	99 ± 3	−21.4 ± 0.4
20-L	5	98 ± 2	105 ± 4	0.128	96 ± 3	−22.1 ± 0.5
30 unloaded	-		82 ± 2	0.100	74 ± 2	−23.9 ± 0.2
30-S	1	99 ± 1	148 ± 1	0.167	100 ± 5	−31.7 ± 0.3
30-M	2	97 ± 2	112 ± 2	0.143	100 ± 4	−24.5 ± 0.3
30-L	5	96 ± 1	113 ± 8	0.122	74 ± 3	−31.6 ± 0.2

**Table 2:** ATR-FTIR band positions (cm^−1^) for liposomal linezolid formulations. Measurements were made in sodium phosphate buffer solutions (pH 7.4, 0.01 M) at 22 °C. The spectrum of the linezolid solution in the corresponding concentration was subtracted as a blank. The SD for each data <0.2 cm^−1^ (*n* = 3).

Sample	ν_as_(CH_2_)	ν_s_(CH_2_)	ν(C=O)	ν_as_(PO_2_^−^)
0 unloaded	2926.0	2853.9	1735.1	1217.1
0-M	2925.0	2853.9	1733.0	1219.6
10 unloaded	2926.9	2854.1	1736.4	1216.1
10-S	2925	2852.7	1731.8	1218.3
10-M	2924.1	2852.7	1731.8	1221.7
10-L	2925.5	2852.7	1731.8	1221.2
20 unloaded	2926.9	2854.1	1733.2	1216.9
20-S	2925.5	2853.2	1730.8	1220.2
20-M	2926	2852.7	1730.8	1219.8
20-L	2925	2853.2	1731.8	1219.8
30 unloaded	2928.4	2851.7	1739.3	1221.1
30-S	2926,5	2852.2	1731.8	1217.8
30-M	2926.5	2852.7	1731.3	1218.8
30-L	2926.9	2852.7	1732.3	1214

## Data Availability

The original contributions presented in this study are included in the article/Supplementary Materials. Further inquiries can be directed to the corresponding author.

## Supplementary Materials

The following supporting information can be downloaded at: https://www.mdpi.com/article/10.3390/pharmaceutics18080994/s1, Figure S1: UV spectrophotometric characterization of linezolid. (A) Chemical structure of linezolid. (B) UV absorption spectrum of 9 μg/mL linezolid solution in a sodium phosphate buffer solution (0.01 M, pH 7.4). (C) Calibration curve used for quantitative determination of linezolid at λ = 250 nm (R^2^ = 0.9981). Figure S2: Stages of tuberculosis granuloma formation in the ex vivo model. Representative bright-field microscopy images (×200 magnification) of granuloma formation at days 5, 8, 9, and 14 of incubation. PBMCs were infected with *M. tuberculosis* H37Rv and cultured in complete RPMI 1640 medium at 37 °C in a 5% CO_2_ atmosphere. Well-formed granulomas (cellular aggregates) were observed starting from days 13–15 and were used for subsequent antibacterial testing. Figure S3: Representative method-specific particle-size distributions obtained for the 10-M liposomal formulation. (A) Intensity-weighted hydrodynamic diameter distribution obtained by dynamic light scattering (DLS). (B) Particle number concentration, as a function of the bin-center diameter, obtained by nanoparticle tracking analysis (NTA). Figure S4: Combined radar plot of the main liposomal ATR-FTIR band shifts (Δν, cm^−1^) for linezolid formulations. The shifts are calculated relative to the corresponding empty liposomes. The plot visualizes the cholesterol-dependent inversion of phosphate hydration and the distinct spectral patterns of formulations with 10%, 20%, and 30% cholesterol. Table S1: Stability of liposomal linezolid formulations upon storage at +4 °C and −20 °C for 1 and 2 months. Parameters monitored: CH_2_ asymmetric stretching band position (ν_as_CH_2_, cm^−1^), drug leakage (%), hydrodynamic diameter (Rh, nm) by DLS, and ζ-potential (mV). Measurements were performed in BS (pH 7.4, 0.01 M) at 22 °C. The ATR-FTIR spectrum of linezolid solution at the corresponding concentration was subtracted as a blank. The SD for each FTIR data point was <0.2 cm^−1^ (*n* = 3). Table S2: Stability of liposomal linezolid formulations upon storage at −20 °C for 1 week with and without cryoprotectants (glucose and mannose). Parameters monitored: CH_2_ asymmetric stretching band position (ν_as_CH_2_, cm^−1^), drug leakage (%), hydrodynamic diameter (Rh, nm) by DLS, and ζ-potential (mV). Measurements were performed in BS (pH 7.4, 0.01 M) at 22 °C. The ATR-FTIR spectrum of linezolid solution at the corresponding concentration was subtracted as a blank. The SD for each FTIR data point was <0.2 cm^−1^ (*n* = 3). Table S3: Coefficients of determination (R^2^) for the kinetic models applied to linezolid release from liposomal formulations in 0.01 M sodium phosphate buffer, pH 7.4, and the same solution supplemented with 1 wt% bovine serum albumin. Models evaluated: zero-order, first-order, Higuchi, and Korsmeyer–Peppas. For Higuchi and Korsmeyer–Peppas models, fitting was performed using data points up to 60% release. Table S4: Korsmeyer–Peppas model parameters (k and n) for linezolid release from liposomal formulations in various media. The model equation is Q = k·t^n^, where Q is the fraction of drug released (%), t is time (min), k is the release rate constant (min^−n^), and n is the release exponent. Fitting was performed using data points up to 60% release.

## Abbreviations

The following abbreviations are used in this manuscript:

ATR-FTIR	Attenuated total reflection Fourier-transform infrared spectroscopy
BSA	Bovine serum albumin
BS	Sodium phosphate buffer solution
CFU	Colony-forming units
CPA	Cryoprotective agent
DLR	Drug-to-lipid ratio
DLS	Dynamic light scattering
EE	Encapsulation efficiency
FBS	Fetal bovine serum
LB	Luria–Bertani medium
Lec	Soy lecithin
LLzd	Liposomal linezolid
Lzd	Linezolid
MIC_app_	Agar diffusion-derived apparent minimum inhibitory concentration
Mtb	*Mycobacterium tuberculosis*
NTA	Nanoparticle tracking analysis
PBMC	Peripheral blood mononuclear cell
PCA	Principal component analysis
PDI	Polydispersity index
RPMI	Roswell Park Memorial Institute medium
SD	Standard deviation
TB	Tuberculosis
UV–Vis	Ultraviolet–visible spectroscopy
WHO	World Health Organization

## References

[1] Chin K.L., Anibarro L., Chang Z.Y., Palasuberniam P., Mustapha Z.A., Sarmiento M.E., Acosta A. (2024). Impacts of MDR/XDR-TB on the global tuberculosis epidemic: Challenges and opportunities. Curr. Res. Microb. Sci..

[2] Ufimtseva E.G., Eremeeva N.I. (2023). Drug-tolerant Mycobacterium tuberculosis adopt different survival strategies in alveolar macrophages of patients with pulmonary tuberculosis. Int. J. Mol. Sci..

[3] Azimi T., Khoshnood S., Asadi A., Heidary M., Mahmoudi H., Kaviar V.H., Hallajzadeh M., Nasiri M.J. (2022). Linezolid resistance in multidrug-resistant mycobacterium tuberculosis: A systematic review and meta-analysis. Front. Pharmacol..

[4] Arbex M.A. (2026). Bedaquiline, Pretomanid, Linezolid, and Moxifloxacin: Mechanisms of Action, Drug Interactions, Adverse Effects and Use in Special Situations. Microorganisms.

[5] Boak L.M., Rayner C.R., Grayson M.L., Paterson D.L., Spelman D., Khumra S., Capitano B., Forrest A., Li J., Nation R.L. (2014). Clinical population pharmacokinetics and toxicodynamics of linezolid. Antimicrob. Agents Chemother..

[6] Romanova M.I., Gaida A.I., Tyulkova T.E., Sergeeva V.I., Le-Deigen I.M., Kolmogorov I.M., Avdeev V.V., Mozhokina G.N., Samoylova A.G. (2026). Linezolid Delivery Methods to the site of tuberculous inflammation: A systematic review. Tuberc. Lung Dis..

[7] Yıldırım M., Düzgüneş N. (2025). Liposome-encapsulated antibiotics for the therapy of mycobacterial infections. Antibiotics.

[8] Fernández-García R., Fraguas-Sánchez A.I. (2024). Nanomedicines for pulmonary drug delivery: Overcoming barriers in the treatment of respiratory infections and lung cancer. Pharmaceutics.

[9] Ponkshe P., Feng S., Tan C. (2021). Inhalable liposomes for treating lung diseases: Clinical development and challenges. Biomed. Mater..

[10] Van Rensburg L., Van Zyl J.M., Smith J. (2018). Deposition and transport of linezolid mediated by a synthetic surfactant Synsurf® within a pressurized metered dose inhaler: A Calu-3 model. Drug Des. Dev. Ther..

[11] Song F., Chen J., Zheng A., Tian S. (2022). Effect of sterols on liposomes: Membrane characteristics and physicochemical changes during storage. LWT.

[12] Papahadjopoulos D., Nir S., Ohki S. (1972). Permeability properties of phospholipid membranes: Effect of cholesterol and temperature. Biochim. Biophys. Acta Biomembr..

[13] Pohle W., Selle C., Fritzsche H., Binder H. (1998). Fourier transform infrared spectroscopy as a probe for the study of the hydration of lipid self-assemblies. I. Methodology and general phenomena. Biospectroscopy.

[14] Paré C., Lafleur M. (1998). Polymorphism of POPE/cholesterol system: A 2H nuclear magnetic resonance and infrared spectroscopic investigation. Biophys. J..

[15] Mannock D.A., Lewis R.N.A.H., McElhaney R.N. (2006). Comparative calorimetric and spectroscopic studies of the effects of lanosterol and cholesterol on the thermotropic phase behavior and organization of dipalmitoylphosphatidylcholine bilayer membranes. Biophys. J..

[16] Briuglia M.L., Rotella C., McFarlane A., Lamprou D.A. (2015). Influence of cholesterol on liposome stability and on in vitro drug release. Drug Deliv. Transl. Res..

[17] Orlikowska-Rzeznik H., Krok E., Chattopadhyay M., Lester C., Piatkowski L. (2024). Cholesterol changes interfacial water alignment in model cell membranes. J. Am. Chem. Soc..

[18] Wallace S.J., Li J., Nation R.L., Rayner C.R., Taylor D., Middleton D., Milne R.W., Boyd B.J. (2013). Physicochemical aspects of the coformulation of colistin and azithromycin using liposomes for combination antibiotic therapies. J. Pharm. Sci..

[19] Kolmogorov I.M., Skuredina A.A., Le-Deygen I.M. (2025). The Drug-to-Lipid-Ratio Determines Fine Structure of the Liposomal Form of Levofloxacin and Molecular Details of Complex Formation with Mannosylated Chitosan. Biochem. Moscow Suppl. Ser. A Membr. Cell Biol..

[20] Johnston M.J., Chang R., Chen H., Lin Y. (2008). Influence of drug-to-lipid ratio on drug release properties and liposome integrity in liposomal doxorubicin formulations. J. Liposome Res..

[21] Klyachko N.L., Haney M.J., Lopukhov A.V., Le-Deygen I.M. (2024). Cationized extracellular vesicles for gene delivery. Sci. Rep..

[22] Rodríguez-Martínez J., Sánchez-Martín M.J., Valiente M. (2023). Efficient controlled release of cannabinoids loaded in γ-CD-MOFs and DPPC liposomes as novel delivery systems in oral health. Microchim. Acta.

[23] Balouiri M., Sadiki M., Ibnsouda S.K. (2016). Methods for in vitro evaluating antimicrobial activity: A review. J. Pharm. Anal..

[24] Filipe V., Hawe A., Jiskoot W. (2010). Critical evaluation of Nanoparticle Tracking Analysis (NTA) by NanoSight for the measurement of nanoparticles and protein aggregates. Pharm. Res..

[25] Yahata S., Hirose M., Ueno T., Nagumo H., Sakai-Kato K. (2021). Effect of Sample Concentration on Nanoparticle Tracking Analysis of Small Extracellular Vesicles and Liposomes Mimicking the Physicochemical Properties of Exosomes. Chem. Pharm. Bull..

[26] Kim A., Ng W.B., Bernt W., Cho N.-J. (2019). Validation of Size Estimation of Nanoparticle Tracking Analysis on Polydisperse Macromolecule Assembly. Sci. Rep..

[27] Magarkar A., Dhawan V., Kallinteri P., Viitala T., Elmowafy M., Róg T., Bunker A. (2014). Cholesterol level affects surface charge of lipid membranes in saline solution. Sci. Rep..

[28] Németh Z., Pallagi E., Dobó D.G., Kozma G., Kónya Z., Csóka I. (2021). An updated risk assessment as part of the QbD-based liposome design and development. Pharmaceutics.

[29] Liu D.Z., Chen W.Y., Tsai L.M., Yang S.P. (2000). The Effects of Cholesterol on the Release of Free Lipids and the Physical Stability of Lecithin Liposomes. J. Chin. Inst. Chem. Eng..

[30] Arsov Z., Quaroni L. (2007). Direct interaction between cholesterol and phosphatidylcholines in hydrated membranes revealed by ATR-FTIR spectroscopy. Chem. Phys. Lipids.

[31] Tai K., Liu F., He X., Ma P., Mao L., Gao Y., Yuan F. (2018). The effect of sterol derivatives on properties of soybean and egg yolk lecithin liposomes: Stability, structure and membrane characteristics. Food Res. Int..

[32] Le-Deygen I.M., Safronova A.S., Mamaeva P.V., Kolmogorov I.M., Skuredina A.A., Kudryashova E.V. (2022). Drug–membrane interaction as revealed by spectroscopic methods: The role of drug structure in the example of rifampicin, levofloxacin and rapamycin. Biophysica.

[33] Moreno M.M., Garidel P., Suwalsky M., Howe J., Brandenburg K. (2009). The membrane-activity of Ibuprofen, Diclofenac, and Naproxen: A physico-chemical study with lecithin phospholipids. Biochim. Biophys. Acta Biomembr..

[34] Boafo G.F., Magar K.T., Ekpo M.D., Qian W., Tan S., Chen C. (2022). The role of cryoprotective agents in liposome stabilization and preservation. Int. J. Mol. Sci..

[35] Susa F., Bucca G., Limongi T., Cauda V., Pisano R. (2021). Enhancing the preservation of liposomes: The role of cryoprotectants, lipid formulations and freezing approaches. Cryobiology.

[36] Suzuki T., Komatsu H. (1996). Effects of glucose and its oligomers on the stability of freeze-dried liposomes. Biochim. Biophys. Acta Biomembr..

[37] Hioki A., Wakasugi A., Kawano K., Hattori Y., Maitani Y. (2010). Development of an in vitro drug release assay of PEGylated liposome using bovine serum albumin and high temperature. Biol. Pharm. Bull..

[38] Gil D., Frank-Kamenetskii A., Barry J., Reukov V., Xiang Y., Das A., Varma A.K., Kindy M.S., Banik N.L., Vertegel A. (2018). Albumin-Assisted Method Allows Assessment of Release of Hydrophobic Drugs from Nanocarriers. Biotechnol. J..

[39] Akaki S., Hosokawa M., Maeda S., Kono Y., Maeda H., Ogawara K.-I. (2024). Efficient Loading into and Controlled Release of Lipophilic Compound from Liposomes by Using Cyclodextrin as Novel Trapping Agent. Biol. Pharm. Bull..

[40] Korsmeyer R.W., Peppas N.A. (1981). Effect of the morphology of hydrophilic polymeric matrices on the diffusion and release of water soluble drugs. J. Membr. Sci..

[41] Korsmeyer R.W., Gurny R., Doelker E., Buri P., Peppas N.A. (1983). Mechanisms of potassium chloride release from compressed, hydrophilic, polymeric matrices: Effect of entrapped air. J. Pharm. Sci..

[42] Alhariri M., Majrashi M.A., Bahkali A.H., Almajed F.S., Azghani A.O., Khiyami M.A., Alyamani E.J., Aljohani S.M., Halwani M.A. (2017). Efficacy of neutral and negatively charged liposome-loaded gentamicin on planktonic bacteria and biofilm communities. Int. J. Nanomed..

[43] Drulis-Kawa Z., Gubernator J., Dorotkiewicz-Jach A., Doroszkiewicz W., Kozubek A. (2006). A comparison of the in vitro antimicrobial activity of liposomes containing meropenem and gentamicin. Cell. Mol. Biol. Lett..

[44] Arrondo J.L.R., Goni F.M., Macarulla J.M. (1984). Infrared spectroscopy of phosphatidylcholines in aqueous suspension a study of the phosphate group vibrations. Biochim. Biophys. Acta Lipids Lipid Metab..

[45] Forge A., Knowles P.F., Marsh D. (1978). Morphology of egg phosphatidylcholine-cholesterol single-bilayer vesicles, studied by freeze-etch electron microscopy. J. Membr. Biol..

[46] Frisken B.J. (2001). Revisiting the Method of Cumulants for the Analysis of Dynamic Light-Scattering Data. Appl. Opt..

[47] Hole P., Sillence K., Hannell C., Maguire C.M., Roesslein M., Suarez G., Capracotta S., Magdolenova Z., Horev-Azaria L., Dybowska A. (2013). Interlaboratory Comparison of Size Measurements on Nanoparticles Using Nanoparticle Tracking Analysis (NTA). J. Nanopart. Res..

[48] McComiskey K.P.M., Tajber L. (2018). Comparison of Particle Size Methodology and Assessment of Nanoparticle Tracking Analysis (NTA) as a Tool for Live Monitoring of Crystallisation Pathways. Eur. J. Pharm. Biopharm..

[49] Malvern Panalytical Characterizing the Size and Concentration of Liposomes Using Multi-Angle Dynamic Light Scattering and Nanoparticle Tracking Analysis. Application Note AN180519, 2018. https://www.malvernpanalytical.com/en/learn/knowledge-center/application-notes/an180519liposomeszetasizernanosight.

[50] Sydykov B., Oldenhof H., de Oliveira Barros L., Sieme H., Wolkers W.F. (2018). Membrane permeabilization of phosphatidylcholine liposomes induced by cryopreservation and vitrification solutions. Biochim. Biophys. Acta Biomembr..

[51] Deniz A., Sade A., Severcan F., Keskin D., Tezcaner A., Banerjee S. (2010). Celecoxib-loaded liposomes: Effect of cholesterol on encapsulation and in vitro release characteristics. Biosci. Rep..

[52] Kaddah S., Khreich N., Kaddah F., Charcosset C., Greige-Gerges H. (2021). Pentacyclic triterpenes modulate liposome membrane fluidity and permeability depending on membrane cholesterol content. Int. J. Pharm..

[53] Santos A., Rodrigues A.M., Sobral A.J.F.N., Monsanto P.V., Vaz W.L.C., Moreno M.J. (2009). Early Events in Photodynamic Therapy: Chemical and Physical Changes in a POPC:Cholesterol Bilayer Due to Hematoporphyrin IX-Mediated Photosensitization. Photochem. Photobiol..

[54] Leite N.B., Martins D.B., Fazani V.E., Vieira M.R., Cabrera M.P.S. (2018). Cholesterol Modulates Curcumin Partitioning and Membrane Effects. Biochim. Biophys. Acta Biomembr..

[55] Subramanian N., Schumann-Gillett A., Mark A.E., O’Mara M.L. (2016). Understanding the Accumulation of P-Glycoprotein Substrates within Cells: The Effect of Cholesterol on Membrane Partitioning. Biochim. Biophys. Acta Biomembr..

[56] Nallamothu R., Wood G.C., Pattillo C.B., Scott R.C., Kiani M.F., Moore B.M., Thoma L.A. (2006). A targeted liposome delivery system for combretastatin A4: Formulation optimization through drug loading and in vitro release studies. PDA J. Pharm. Sci. Technol..

[57] Roy A., Datta S., Bhattacharya S., Dasgupta S. (2018). Underlying molecular interaction of bovine serum albumin and linezolid: A biophysical outlook. J. Biomol. Struct. Dyn..

[58] Zborowski J., Roerdink F., Scherphof G. (1977). Leakage of sucrose from phosphatidylcholine liposomes induced by interaction with serum albumin. Biochim. Biophys. Acta Gen. Subj..

[59] Bonté F., Juliano R.L. (1986). Interactions of liposomes with serum proteins. Chem. Phys. Lipids.

[60] Dimitrova M.N., Matsumura H., Dimitrova A., Neitchev V.Z. (2000). Interaction of albumins from different species with phospholipid liposomes. Multiple binding sites system. Int. J. Biol. Macromol..

[61] Zambito Y., Pedreschi E., Di Colo G. (2012). Is dialysis a reliable method for studying drug release from nanoparticulate systems?—A case study. Int. J. Pharm..

[62] Fugit K.D., Anderson B.D. (2014). Dynamic, Nonsink Method for the Simultaneous Determination of Drug Permeability and Binding Coefficients in Liposomes. Mol. Pharm..

[63] Harish N.M., Kiran A.B., Rathnanand M., Shirwaikar A., Shenoy K.R.P. (2007). Sustained-release matrix tablets of terbutaline sulphate: Formulation and in vitro evaluation. Indian Drugs.

[64] Ritger P.L., Peppas N.A. (1987). A simple equation for description of solute release I. Fickian and non-fickian release from non-swellable devices in the form of slabs, spheres, cylinders or discs. J. Control. Release.

[65] Wu I.Y., Bala S., Skalko-Basnet N., Di Cagno M.P. (2019). Interpreting non-linear drug diffusion data: Utilizing Korsmeyer-Peppas model to study drug release from liposomes. Eur. J. Pharm. Sci..

