# Supplementary material for: Membrane Interfacial Organization Determines the Functional Performance of Liposomal Linezolid
Source: Pharmaceutics. 2026 Aug 11;18(8):994. doi: 10.3390/pharmaceutics18080994 (PMC13516561; doi:10.3390/pharmaceutics18080994)
Supplement: Supplementary file 1 [file pharmaceutics-18-00994-s001.zip › pharmaceutics-4442711-supplementary.pdf]

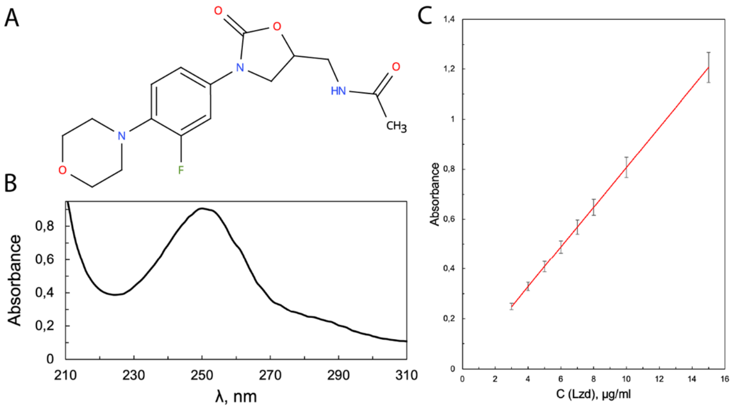

Figure S1. *UV spectrophotometric characterization of linezolid. A Chemical structure of linezolid. B UV absorption spectrum of 9 µg/ml linezolid solution in phosphate-buffered saline (0.01 M, pH 7.4). C Calibration curve used for quantitative determination of linezolid at λ = 250 nm (R^2 = 0,9981)*

Day 5 of granuloma formation

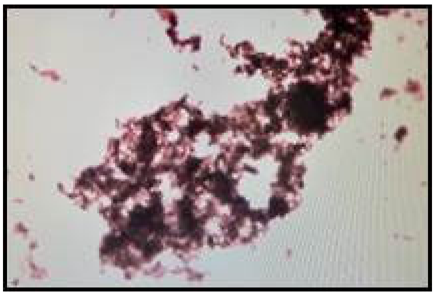

Day 8 of granuloma formation

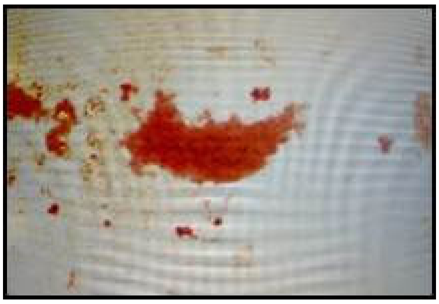

Day 9 of granuloma formation

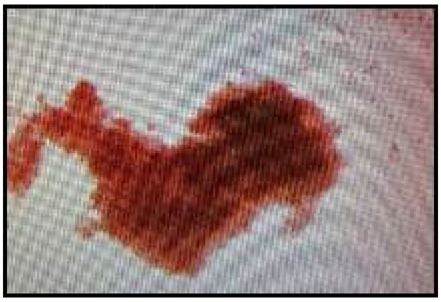

Day 14 of granuloma formation

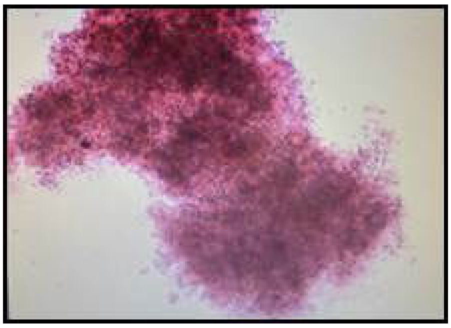

Figure S2. Stages of tuberculosis granuloma formation (×200). On the day after treatment, the contents of the wells were sampled and plated onto selective Middlebrook 7H10 agar (Himedia, India) to quantify viable mycobacteria. The plates were incubated for 21 days under standard conditions, and then colony-forming units (CFU) were enumerated.

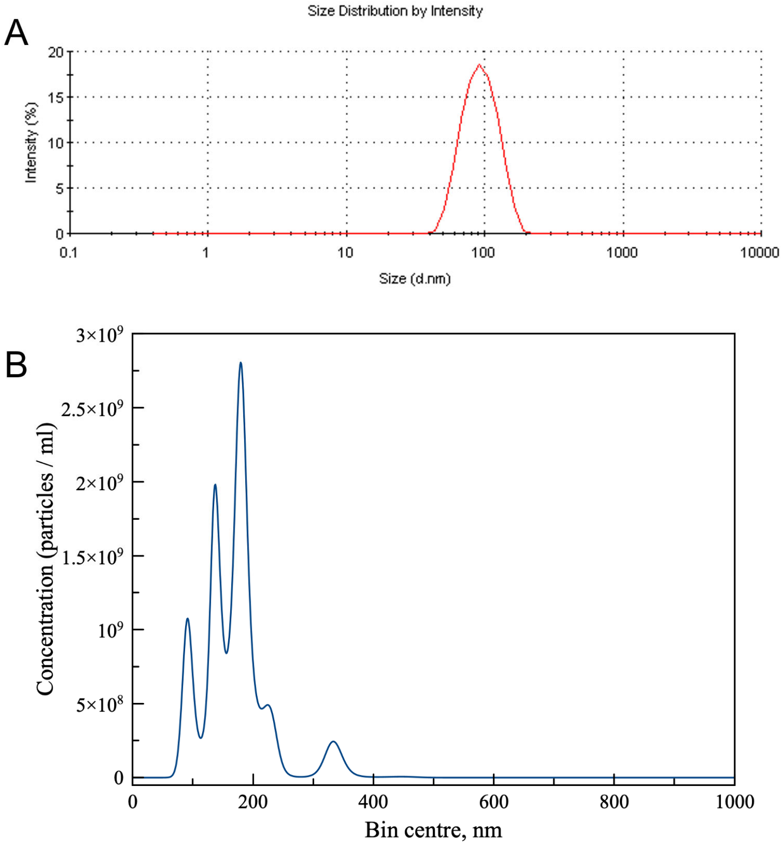

Figure S3. Representative method-specific particle-size distributions obtained for the 10-M liposomal formulation. (A) Intensity-weighted hydrodynamic diameter distribution obtained by dynamic light scattering (DLS). (B) Particle number concentration as a function of bin-center diameter obtained by nanoparticle tracking analysis (NTA).

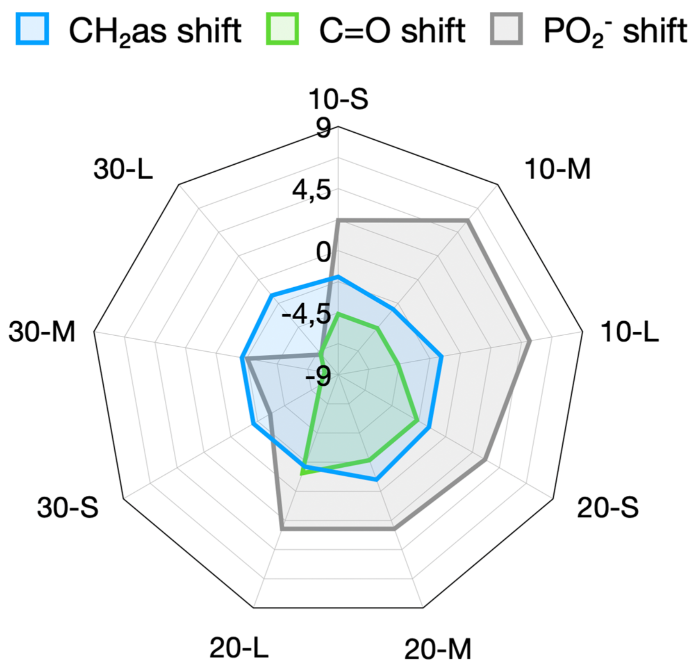

Figure S4. Combined radar plot of the main liposomal ATR-FTIR band shifts (Δν, cm^{-1}) for linezolid formulations. The shifts are calculated relative to the corresponding empty liposomes.

Table S1. Stability of liposomal linezolid formulations upon storage at +4 °C and –20 °C for 1 and 2 months. Measurements in BS (pH 7.4, 0.01 M) at 22 °C. The ATR-FTIR spectrum of linezolid solution in the corresponding concentration was subtracted as a blank. The SD for each FTIR data < 0.2 cm^{-1} (n = 3).

Sample	CH_2as	% Leakage	Rh, nm (DLS)	ζ-potential, mV
		1 month storage, +4C		
10-S	2926,0	5,38%	59	-16,2
10-M	2926,9	2,37%	64	-17,3
10-L	2926,2	0,10%	57	-13,6
20-S	2927,2	0,10%	85	-25,6
20-M	2927,7	6,65%	70	-19,7
20-L	2927,4	2,70%	74	-16,4
30-S	2928,7	6,93%	208	-18,2
30-M	2928,7	0,10%	91	-19,2
30-L	2927,7	1,27%	72	-16,4
		2 months storage, +4C		
10-S	2923,6	8,55%	67	-20,9
10-M	2924,1	4,65%	59	-18,2
10-L	2923,6	0,99%	56	-8,7
20-S	2924,5	0,10%	86	-20,7
20-M	2924,1	8,54%	71	-18,2
20-L	2923,6	2,80%	58	-11,1
30-S	2925,5	7,03%	207	-24,3
30-M	2925,0	0,10%	93	-21,9
30-L	2924,5	1,37%	78	-10,5
		1 month storage, -20C		
10-S	2930,5	6,24%	80	-23,6
10-M	2932,3	6,43%	97	-27,2
10-L	2933,4	0,07%	86	-20,2
20-S	2931,3	8,58%	187	-29,4
20-M	2930,5	12,87%	164	-30,0
20-L	2930,5	4,68%	92	-21,0

30-S	2931,3	14,77%	1235	-31,4
30-M	2933,4	10,10%	233	-30,6
30-L	2927,4	2,26%	109	-18,2
		2 months storage, -20C		
10-S	2925,0	7,96%	109	-20,5
10-M	2925,0	9,30%	84	-22,5
10-L	2925,0	0,10%	93	-21,6
20-S	2925,0	9,95%	282	-27,0
20-M	2924,5	23,86%	145	-24,7
20-L	2925,0	4,93%	106	-17,5
30-S	2925,0	27,32%	869	-26,9
30-M	2925,0	16,32%	206	-26,7
30-L	2924,5	2,26%	112	-21,1

Table S2. Stability of liposomal linezolid formulations upon storage –20 °C for 1 week with and without cryoprotectants. Measurements in BS (pH 7.4, 0.01 M) at 22 °C. The ATR-FTIR spectrum of linezolid solution in the corresponding concentration was subtracted as a blank. The SD for each FTIR data < 0.2 cm^{-1} (n = 3).

Sample	% Leakage	Rh, nm (DLS)	ζ-potential, mV
		No cryoprotectants	
10-S	4,31%	81	-23,3
20-S	12,45%	122	-27,0
30-S	11,07%	524	-28,0
		Glucose	
10-S	3,83%	79	-20,3
20-S	9,84%	107	-21,5
30-S	11,85%	179	-25,4
		Mannose	
10-S	4,24%	74	-19,5
20-S	9,06%	100	-22,6
30-S	12,60%	155	-24,2

Table S3. Coefficients of determination (R^2) for the kinetic models applied to linezolid release from liposomal formulations in 0,01 M sodium-phosphate buffer solution, pH 7,4 (BS) and the same solution supplemented with bovine serum albumin, 1 w.% (+BSA)

Medium	Formulation	Zero-order R^2	First-order R^2	Higuchi R^2	Korsmeyer–Peppas R^2
BS	10-S	0.9030	0.8016	0.7154	0.9879
BS	10-M	0.9044	0.8662	0.7171	0.9941
BS	10-L	0.9013	0.8738	0.7135	0.9972
BS	20-S	0.8987	0.8722	0.7107	0.9960
BS	20-M	0.9035	0.8787	0.7165	0.9983
BS	20-L	0.9032	0.8819	0.7160	0.9988
BS	30-S	0.9025	0.8918	0.7143	0.9950
BS	30-M	0.9011	0.8463	0.7127	0.9940
BS	30-L	0.9033	0.8818	0.7158	0.9981
BSA	10-S	0.9132	0.7368	0.7317	0.9875
BSA	10-M	0.9068	0.7371	0.7199	0.9913
BSA	10-L	0.9123	0.8765	0.7265	0.9912
BSA	20-S	0.9498	0.8497	0.8228	0.9973

BSA	20-M	0.9711	0.8223	0.8527	0.9935
BSA	20-L	0.9524	0.7184	0.8367	0.9971
BSA	30-S	0.9474	0.7859	0.8649	0.9936
BSA	30-M	0.8999	0.6781	0.7122	0.9899
BSA	30-L	0.9232	0.7106	0.7411	0.9817

Table S4. Korsmeyer–Peppas parameters (k, n) for linezolid release from liposomes in 0,01 M sodium-phosphate buffer solution (BS) and with 1% BSA (BSA) . Fitting was performed up to 60% release.

Medium	Sample	n
BS	10 L	1.9176
BS	10 M	1.8714
BS	10 S	1.8017
BS	20 L	1.9371
BS	20 M	1.9166
BS	20 S	1.8848
BS	30 L	1.9195
BS	30 M	1.8720
BS	30 S	1.8947
BSA	10 L	1.8039
BSA	10 M	1.8040
BSA	10 S	1.7550
BSA	20 L	1.9190
BSA	20 M	1.8573
BSA	20 S	1.9518
BSA	30 L	2.1415
BSA	30 M	1.7889
BSA	30 S	1.8386
